# Investigation into the effect of phenylalanine gating on anaerobic haem breakdown using the energy landscape approach

**DOI:** 10.1002/pro.5243

**Published:** 2025-01-28

**Authors:** Alasdair D. Keith, Elizabeth B. Sawyer, Desmond C. Y. Choy, James L. Cole, Cheng Shang, George S. Biggs, Oskar James Klein, Paul D. Brear, David J. Wales, Paul D. Barker

**Affiliations:** ^1^ Yusuf Hamied Department of Chemistry University of Cambridge Cambridge UK; ^2^ Department of Biochemistry, Sanger Building University of Cambridge Cambridge UK

**Keywords:** biophysics, energy landscapes, heme, HemS, nicotinamide adenine dinucleotide (NADH), protein dynamics, protein gates, residue‐mediated regulation, *Yersinia enterocolitica*

## Abstract

We have recently demonstrated a novel anaerobic NADH‐dependent haem breakdown reaction, which is carried out by a range of haemoproteins. The *Yersinia enterocolitica* protein, HemS, is the focus of further research presented in the current paper. Using conventional experimental methods, bioinformatics, and energy landscape theory (ELT), we provide new insight into the mechanism of the novel breakdown process. Of particular interest is the behavior of a double phenylalanine gate, which opens and closes according to the relative situations of haem and NADH within the protein pocket. This behavior suggests that the double phe‐gate fulfills a regulatory role within the pocket, controlling the access of NADH to haem. Additionally, stopped‐flow spectroscopy results provide kinetic comparisons between the wild‐type and the selected mutants. We also present a fully resolved crystal structure for the F104AF199A HemS monomer, including its extensive loop, the first such structure to be completely resolved for HemS or any of its close homologues. The energy landscapes approach provided key information regarding the gating strategy employed by HemS, compensating for current limitations with conventional biophysical and molecular dynamics approaches. We propose that ELT become more widely used in the field, particularly in the investigation of the dynamics and interactions of weak‐binding ligands, and for gating features, within protein cavities.

## 1 INTRODUCTION

Catabolism of exogenous haem by bacterial pathogens is typically mediated by a haem oxygenase (HO) via a process requiring three O_2_ molecules, and ultimately yielding regiospecific biliverdin and CO alongside the extracted Fe^2+^ ion (Lyles and Eichenbaum 2018; Maines and Kappas 1974; Unno et al. 2007; Wilks and Heinzl 2014; Wilks and Ikeda‐Saito 2014). Many bacterial pathogens are facultatively anaerobic and can thus thrive under oxygen‐depleted conditions (André et al. 2021; Bottone 1997; Gleason and Patterson 1982; Griffin et al. 1990; Hale and Keusch 1996; Hentges 1996; Le Baut et al. 2018; Mattock and Blocker 2017; Pedraz et al. 2020; Shoaib et al. 2019), where access to this method of haem breakdown is therefore more limited.

Our previous report (Keith et al. 2023) discusses a novel anaerobic, nicotinamide adenine dinucleotide (NADH)‐dependent haem breakdown process catalyzed by a family of enzymes, which are each thought to be the end‐users of a sophisticated haem uptake and processing strategy specifically evolved to operate under oxygen‐deficient conditions. We argued in that report that this strategy helps to explain the survival of various pathogens within endorthermic oxygen‐limited organs, including *Yersinia enterocolitica*, *Yersinia pestis*, *Escherichia coli* (O157:H7), and *Shigella dysenteriae*.

Within this family of enzymes, HemS, from *Y. enterocolitica*, is the focus of the present study. *Yersinia enterocolitica* is a facultative anaerobic Gram‐negative pathogenic bacterium, which typically enters endotherms via the gastrointestinal tract. It is the strain chiefly responsible for yersiniosis and, though symptoms tend to be relatively benign, more serious conditions such as endocarditis (Karachalios et al. 2002), acute gastroenteritis (Saebø and Lassen 1992), mesenteric lymphadenitis (Zinczuk et al. 2015), and fulminant septicemia (Reinicke and Korner 1977) can develop.

The HemS haemoprotein has already been investigated and the *apo*‐ (PDB: 2J0R) (Schneider et al. 2006) and *holo*‐ (PDB: 2J0P) (Schneider and Paoli 2005) crystal structures solved. These studies showed that the 41 kDa HemS molecule consists of two topologically homologous domains connected via an unstructured loop, thus yielding a central pair of stacked *β*‐sheets. These *β*‐sheets are capped by *α*‐helices, forming two distinct pockets. Within the larger of these pockets is the primary haem‐binding site, which binds to a histidine residue (H196) via one of its free iron axial sites, thus anchoring haem inside the pocket. This bond to H196 causes significant buckling and distortion to the porphyrin ring, suggesting that haem is being primed for degradation.

The function(s) of the cytosolic “S” family of haemoproteins, of which HemS is a member, has long been debated. In their original study of the *hem* operon, Stojiljkovic and Hantke deduced that HemS “could be either a cytoplasmic membrane permease that transfers hemin into the cytoplasm or a hemin‐degrading enzyme” (Stojiljkovic and Hantke 1992). Since then, PhuS, which has 42.6% sequence identity with HemS, has been demonstrated to act as a haem chaperone to the bona fide haem oxygenase, HemO (Lansky et al. 2006). However, the situation of histidine residues within the PhuS pocket are not analogous to the situation in HemS, or the latter's closer homologues, such as HmuS (from *Yersinia pestis*) and ChuS (from *Escherichia coli* O157:H7). PhuS contains two additional histidine residues (H210 and H212) in close proximity to the axially haem‐binding residue, H209, which are more exposed to the cavity opening, thus facilitating haem transfer, which is a structural feature not present in HemS, nor HmuS or ChuS. Studies on ChuS suggest this homologue can engage both in haem transfer and breakdown. Under aerobic conditions, haem degradation to a novel product has been observed following the reaction of *holo*‐ChuS with ascorbic acid (Suits et al. 2005). With the previous lack of evidence for haem breakdown under anaerobic conditions, older studies proposed that ChuS acts as a haem storage protein when oxygen is limited, ultimately transferring haem to ChuW for breakdown (Mathew et al. 2019). However, our previous report demonstrates that ChuS, HemS, and a number of other haemoproteins within this family engage in anaerobic haem breakdown using NAD(P)H. Structural formulae for haem and NAD(P)H are given in Figure S1, Supporting Information.

This research demonstrated that breakdown begins with hydride transfer from NAD(P)H to haem and that the novel breakdown product is cleaved at one of the *meso*‐carbon positions. This cleavage is presumably in order to facilitate extraction of the iron ion. It was unclear to us whether NADH or NADPH was the natural ligand as they appear to interact identically with *holo*‐HemS and computations suggested they dock to the same position. A practical decision was therefore made to consider NADH in further investigations. We found that this reaction can proceed under both aerobic and anaerobic conditions and that the latter is likely the natural, purposive condition, since the presence of oxygen leads to the formation of an additional product (non‐regiospecific biliverdin) arising from a non‐enzymatic process, coupled oxidation (Lemberg 1935; Warburg and Negelein 1930; Wilks and Ikeda‐Saito 2014).

Further insight into the mechanism of this novel reaction within HemS was gained through a detailed mutagenesis study. Investigations into the residues involved in direct axial binding to the iron ion of haem proved to be straightforward, and provided key insight into those residues necessary for haem to bind securely inside the pocket, and hence facilitate the haem breakdown reaction. Study of the residues involved in NADH‐binding proved to be more difficult. Since NADH was involved in a novel mode of binding, there is very little data to compare with in the literature, and the transient binding made characterization difficult with traditional biophysical techniques.

**FIGURE 1:**
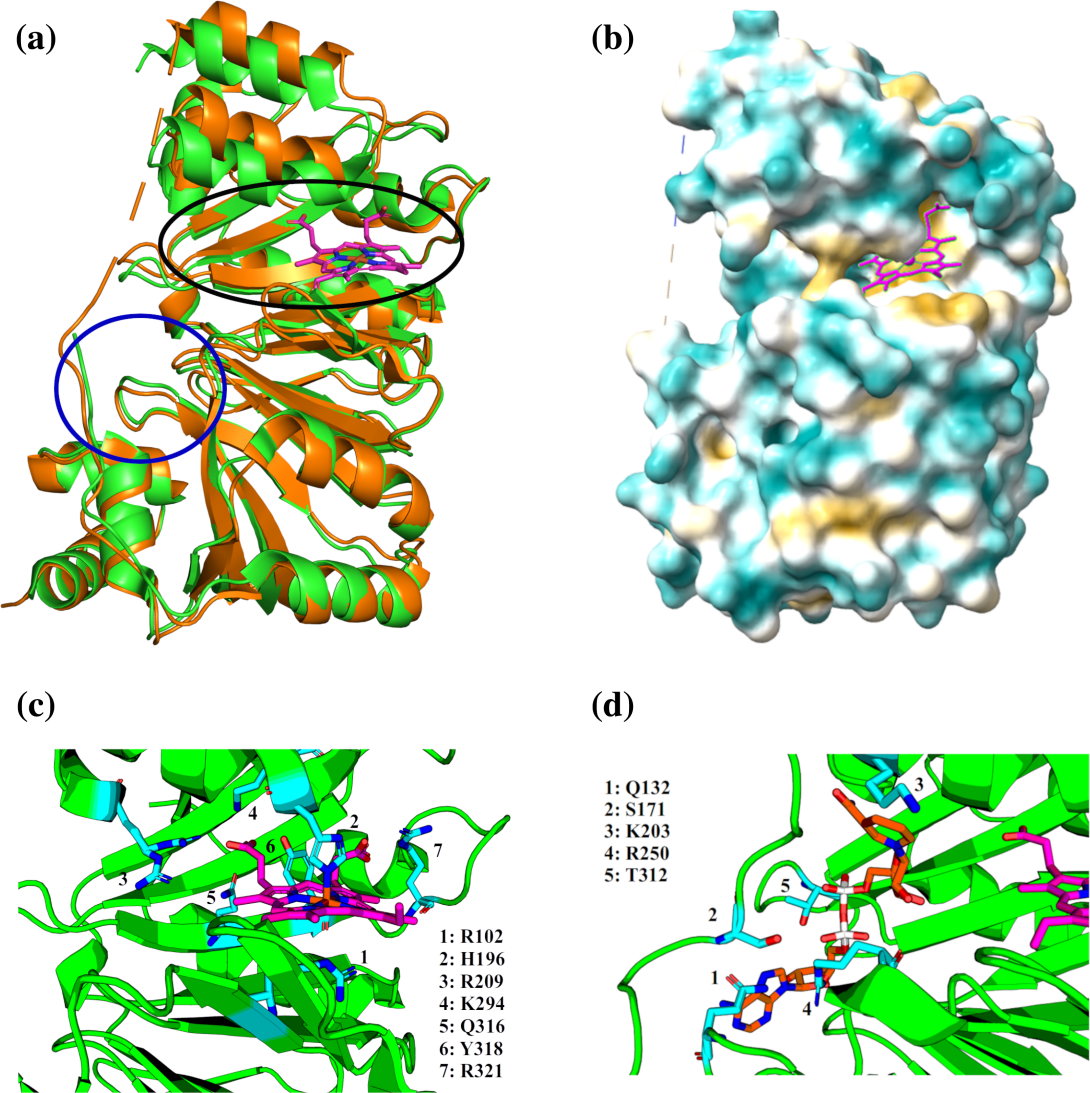
HemS structure and ligand binding sites. (a) *holo*‐HemS (PDB: 2J0P, green) superimposed on *apo*‐HemS (PDB: 2J0R, burnt orange). Haem from the *holo*‐structure is shown in magenta. The black circle corresponds to the large cavity, and the blue circle to the small cavity. The unstructured loop is entirely missing from the *holo*‐structure and incomplete in the *apo*‐structure. The structural overlay clearly demonstrates the clamping effect HemS undergoes upon haem inclusion. (b) Space‐filling model of *holo*‐HemS, as calculated by Chimera (Pettersen et al. 2004). Residues are color‐coded, with cyan indicating hydrophilicity and yellow hydrophobicity. (c) Magnified representation of haem in the HemS pocket, with haem‐binding residues numbered and highlighted in cyan. (d) Magnified representation of NADH (orange) in the HemS pocket. Residues directly interacting with NADH are explicitly represented in cyan.

In our previous publication (Keith et al. 2023) we identified NADH‐binding modes within HemS. Relibase^+^ (Hendlich 1998), a now‐retired bioinformatics package designed to identify unexpected resemblances between protein cavities while being mainly independent of sequence‐ and fold‐homology, was used to identify structurally similar NADH‐binding sites. This search only yielded weak “hits,” emphasizing the uniqueness of this HemS protein cavity interaction with NADH. Nevertheless, the strongest of these hits suggested a possible, stretched conformation for NADH to access haem in the HemS pocket. After superimposing this NADH structure onto the coordinates for *holo*‐HemS, calculations were performed, first to identify further possible binding sites within the pocket, and then to connect these sites via transition states, thus generating extended pathways. The most important residues implicated in direct NADH‐binding by these calculations are shown in Figure 1d, which is reproduced from our previous publication (Keith et al. 2023). Q132, S171, and R250 all interact with the adenine base of NADH, while the latter additionally interacts with the proximate adenosine ribose oxygen and phosphate group. K203 interacts with the amide of the nicotinamide head, perhaps suggesting a role for this residue in guiding this head towards haem. T312, meanwhile, interacts with the hydroxyl groups of the adenosine ribose as well as the phosphate groups.

Upon inspecting the published crystal structures for HemS (PDB codes 2J0P (Schneider et al. 2006) and 2J0R (Schneider and Paoli 2005)), we noticed a potential double phenylalanine gate (comprising residues F104 and F199) between the haem‐binding site and the purported NADH‐binding site. Such gates, which are often aromatic (Zhou and McCammon 2010), are widely known to regulate inter‐ligand activities within protein cavities (Gora et al. 2013; McCammon and Northrup 1981; Zhou et al. 1998), but their accurate study is notoriously difficult (Arroyo‐Mañez et al. 2011; Gora et al. 2013). Standard molecular dynamics (MD) applications typically do not adequately account for the kinetic profiles of such regulatory features within proteins (Gora et al. 2013), as they omit true transition states by applying Newtonian approximations. In other words, standard MD, though often excellent at estimating ligand modes of binding when there are few barriers to entry and/or to movement within the protein cavity, does not typically perform well when significant barriers exist (Bernardi et al. 2015), as is the case when the protein cavity contains a gate or other regulatory feature. Furthermore, such gating schemes can lead to extended ligand–ligand interaction timescales, thus introducing an often intractable number of errors to standard MD simulations (Elber 2016). Though various enhanced sampling methods have been developed to overcome these limitations (Earl and Deem 2005; Hamelberg et al. 2004; Sugita and Okamoto 1999; Torrie and Valleau 1977; Wang and Landau 2001), their typical application is in the prediction of thermodynamic, rather than kinetic, properties (Joseph et al. 2017). The identification of key intermediates along multiple competing pathways has proven to be a more difficult problem to solve, with rare events sampling techniques, such as transition path sampling (Bolhuis et al. 2002; Dellago et al. 1998) and forward flux sampling (Allen et al. 2009), typically being computationally expensive, and difficult to keep both unbiased and uncorrelated (Chong et al. 2017).

By contrast, the computational energy landscape approach (Wales 2003; Wales 2012; Wales 2018; Wales et al. 2000; Wales and Bogdan 2006), as applied to multiple previous biochemical studies (Joseph et al. 2017; Klenin et al. 2011; Onuchic et al. 1997; Wolynes 2005), can treat rare events and long experimental time scales within a well‐defined set of approximations (Burke et al. 2020; Klenin et al. 2011; Wales 2003; Wales 2005; Wales 2010). Here we consider the energy landscape in terms of local minima and the transition states that connect them in a kinetic transition network (Noé and Fischer 2008; Prada‐Gracia et al. 2009; Wales 2010). The landscape is visualized using disconnectivity graphs (Becker and Karplus 1997; Wales et al. 1998), as their underlying structures can provide qualitative thermodynamic and kinetic insight (Calvo et al. 2000; Doye et al. 1998; Doye et al. 1999; Frantsuzov and Mandelshtam 2005; Liu and Jordan 2005; Neirotti et al. 2000; Predescu et al. 2005; Sharapov et al. 2007; Sharapov and Mandelshtam 2007; Wales 2003; Wales and Doye 1997). A detailed discussion of disconnectivity graphs, which may be unfamiliar to the reader, is provided in the methods. Postprocessing using statistical mechanics and unimolecular rate theory also allows for the quantitative analysis of global thermodynamics and kinetics (Noé and Fischer 2008; Prada‐Gracia et al. 2009; Rao and Caflisch 2004; Wales 2010).

Due to these advantages, we applied the energy landscapes approach to investigate the behavior of the double phenylalanine (phe‐) gate within HemS, in order to resolve its regulatory mechanism to high resolution. We then tested our predictions by applying standard biophysical techniques to relevant mutated variants of recombinantly expressed HemS. UV–visible and stopped‐flow spectroscopy demonstrated that these mutants were capable of carrying out the novel anaerobic haem breakdown reaction, albeit to a limited extent, in agreement with the theoretical predictions. Together, these results provide key insight into the behavior of the phe‐gate, where it appears to engage in self‐regulation of haem breakdown. Furthermore, crystallographic analysis of F104AF199A HemS (i.e., HemS with this double phe‐gate removed) provided key insight into HemS structural stability and is the first fully resolved monomer structure within this class of haemoprotein. As demonstrated by this study, we envisage that the energy landscapes approach could potentially support investigations into numerous biochemical regulatory mechanisms contingent on complex gating schemes.

## 2 RESULTS AND DISCUSSION

### 2.1 Double phenylalanine gate

Though not directly involved in binding to haem, we hypothesized the double‐phe gate to be of both structural and functional importance due to its position at the center of the cavity, separating haem from NADH. We therefore carefully selected mutants to study, as detailed in the methods and shown in Figure S2 and Table S1. A phylogenetic study of the wider family to which HemS belongs showed that this double phe‐gate was very well conserved, unlike the residues implicated in NADH‐binding. Taken together with the highly conserved haem‐binding residues, as shown in Table S2, these data suggested that all of the homologues within this wider family are haemoproteins, but that not all of them are haem breakdown enzymes, or at least not all of them use NADH to bring about such breakdown. We therefore hypothesize that the double phe‐gate can regulate access of a range of biomolecules to haem and is not just limited to NADH. The full details of the phylogenetic study can be found in our previous report (Keith et al. 2023), with the phylogenetic tree reproduced in Figure S3.

**FIGURE 2:**
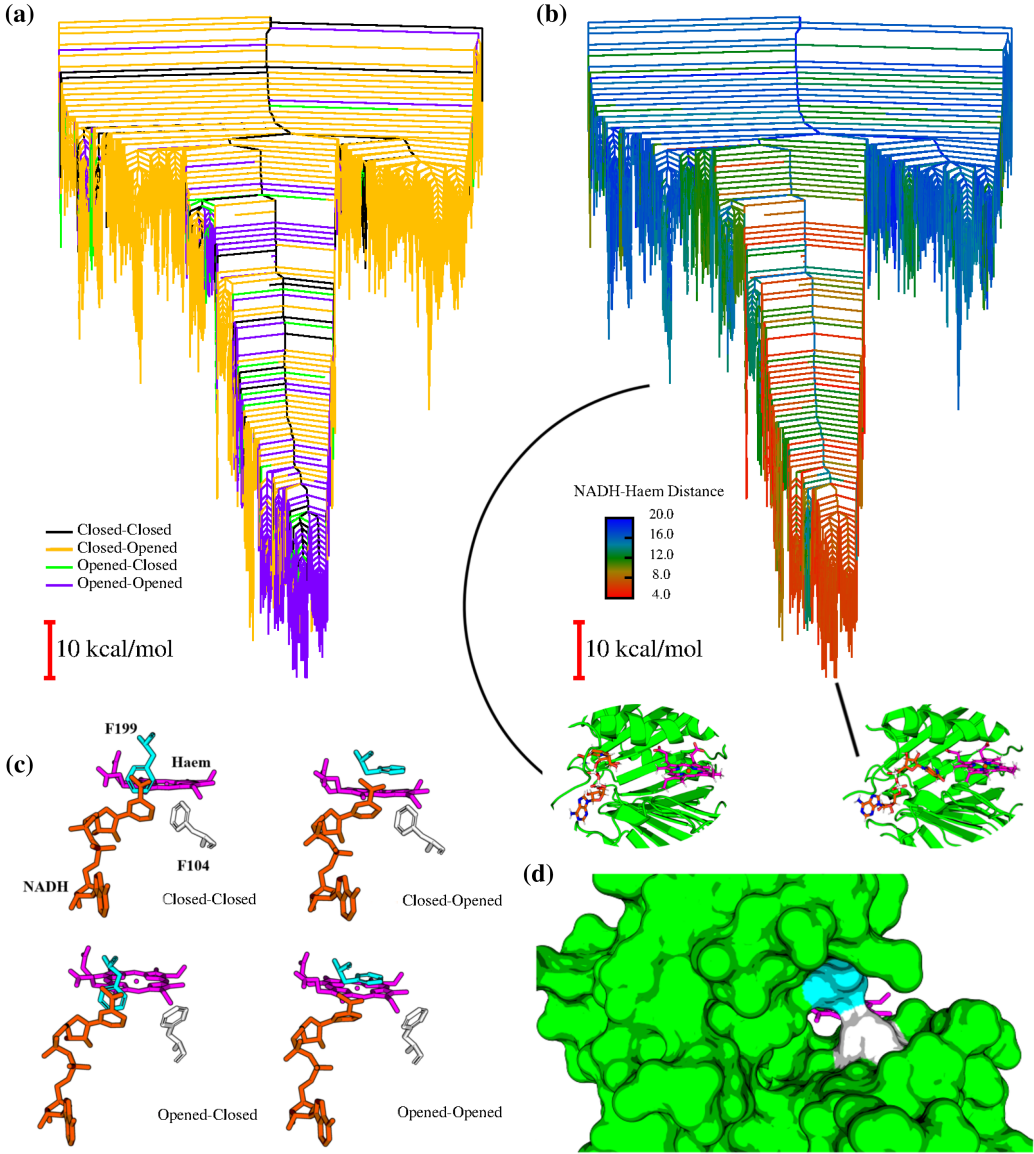
Double phe‐gate regulation of NADH access to haem. (top) Disconnectivity graphs depicting the energy landscape for WT HemS, haem and NADH. (a) Graph is color‐coded according to the conformations of the double phe‐gate residues, F104 and F199. See the methods for definitions of the opened and closed states. (b) Graph is color‐coded according to NADH–haem distance, given in Å. Two minima are highlighted, with their magnified images focused on the main cavity. The double phe‐gate is not explicitly shown to avoid crowding the visualization of the pocket. (c) Representations of possible double phe‐gate conformations. All protein atoms other than those depicting the gates have been removed for clarity. (d) Space‐filling representation of a fully closed double phe‐gate, from the perspective of NADH as it enters the pocket.

Figure 2c shows the different conformations the double phe‐gate (which comprise the F104 and F199 residues) can adopt. Clearly, NADH is prevented from accessing haem in the closed‐closed case, and access is limited when just one of the two gates are open. Figure 2d, which is from the viewpoint of the NADH molecule, shows even more clearly that a closed‐closed double phe‐gate prevents access to haem via a space‐filling representation.

Disconnectivity graphs are a useful method of depicting complex, high‐dimensional energy landscapes. For a detailed introduction, please see the methods. The disconnectivity graphs plotted in Figure 2 reveal a close relationship between the double phe‐gate conformation, and the proximity of NADH to haem. The graphs depict the same landscape, with (a) color‐coded according to the double phe‐gate conformation within the minima plotted, and (b) color‐coded according to the NADH–haem distance. The landscape shows clear funneling properties, with little frustration that would hinder the system from reaching low‐lying configurations. These properties are typical of evolved biomolecular systems. For example, protein‐folding landscapes typically have well‐defined funnels, suggesting that the protein represented by the landscape folds easily and accurately to its native state (Carr and Wales 2008; Joseph et al. 2019; Neelamraju et al. 2018; Prentiss et al. 2010). In the present case, the protein is already at, or near to, its optimal fold, and the landscape instead represents the energetic profile of the protein, its two ligands and their relative conformations and situations all considered with respect to one another. Here, a deep, well‐defined funnel indicates that there is a strong energetic driving force to bind the two ligands involved in the system at well‐defined sites and in particular orientations. The fact that the bottom of the funnel strongly correlates with short NADH–haem distances shows that these optimal configurations have the two ligands in close proximity, most likely to react with one another. The two highlighted minima in Figure 2b show this correlation: whereas the high‐energy minimum outside the main funnel has NADH folded and far from haem, the most stabilized minimum at the bottom of the funnel has NADH fully stretched out and in close proximity to the other ligand. This stabilization is remarkable given that NADH, when free in solution, preferentially occupies a folded conformation (Patel 1969; Weber 1957), with unfolding requiring intramolecular hydrogen bond breaking. The graph plotted in Figure 2a shows that the lower‐energy states correspond to both of the gates being open, and comparing the graphs reveals that states with short NADH–haem distances tend to have both gates open, whereas those states not found in the main funnel typically have a closed F104 gate and open F199 gate. Clearly, when NADH is close to the double phe‐gate, opening the entire gate allows NADH to pass through more easily to haem. In that sense, the correlation between NADH–haem distance and double phe‐gate conformation is not surprising. The prevalence of the closed‐opened conformation in minima outside the base of the main funnel suggests that this is a particularly stable conformation when NADH is folded and at the edge of the cavity. As shown in Figure 2c, this conformation features the two phenylalanines engaged in a T‐shaped *π*–*π* bonding interaction. At some point, F104 then flips to an opened conformation as NADH unfolds and approaches haem; this change allows for the avoidance of steric crowding at the center of the cavity. The closed‐closed and opened‐closed conformations, though occasionally achieved, are not significant competitors.

### 2.2 Comparing energy landscapes with and without NADH

**FIGURE 3:**
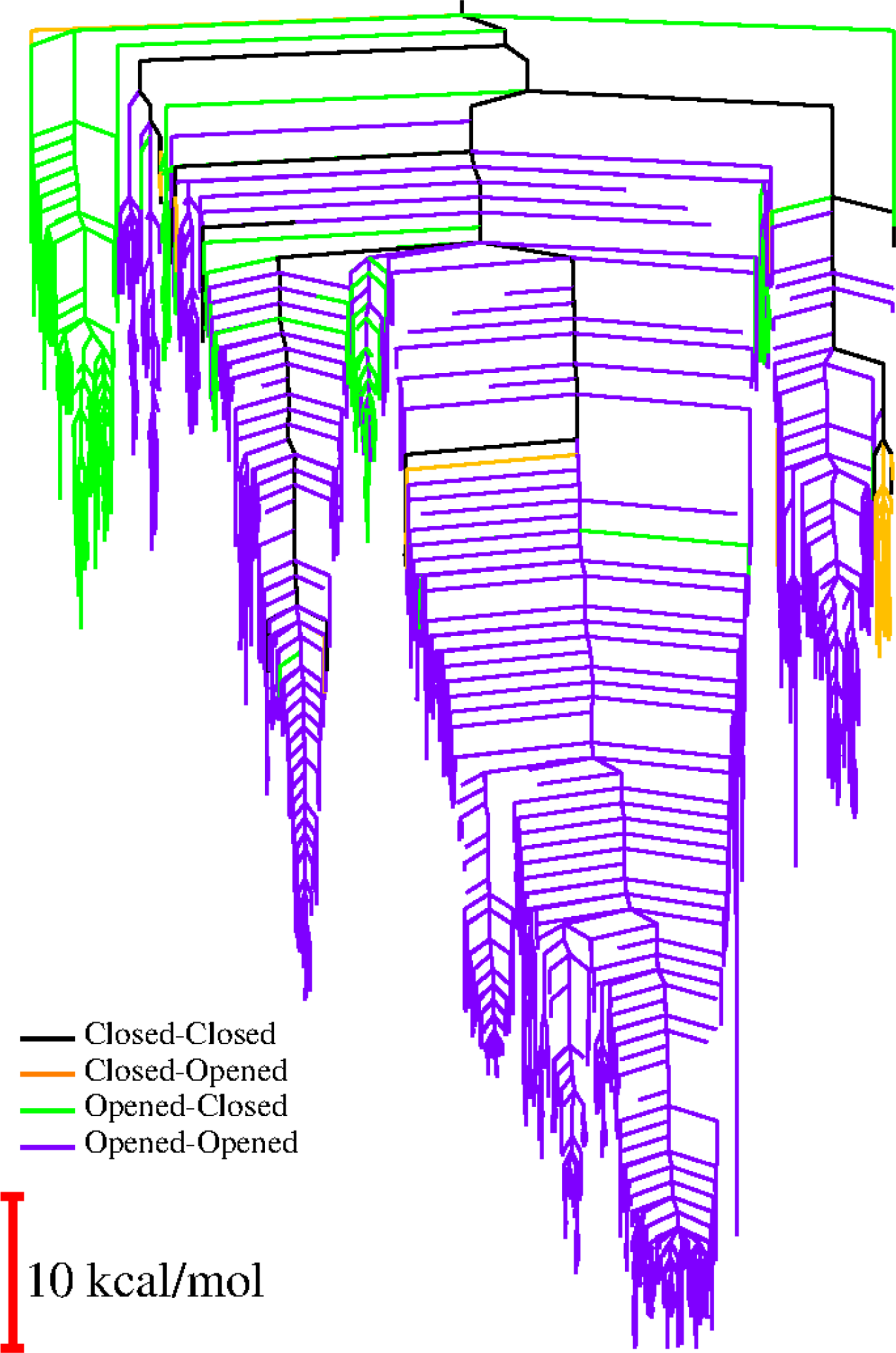
Disconnectivity graph of HemS + haem, color‐coded according to double phe‐gate conformation. The database is not as complete as the database of the system that includes NADH; nevertheless, general properties can still be identified. Comparison against the disconnectivity graph where NADH is present shows that, in the absence of NADH, minima typically favor both gates being opened, whereas when NADH is included, the closed‐opened conformation becomes most prevalent other than when NADH is close to haem.

**FIGURE 4:**
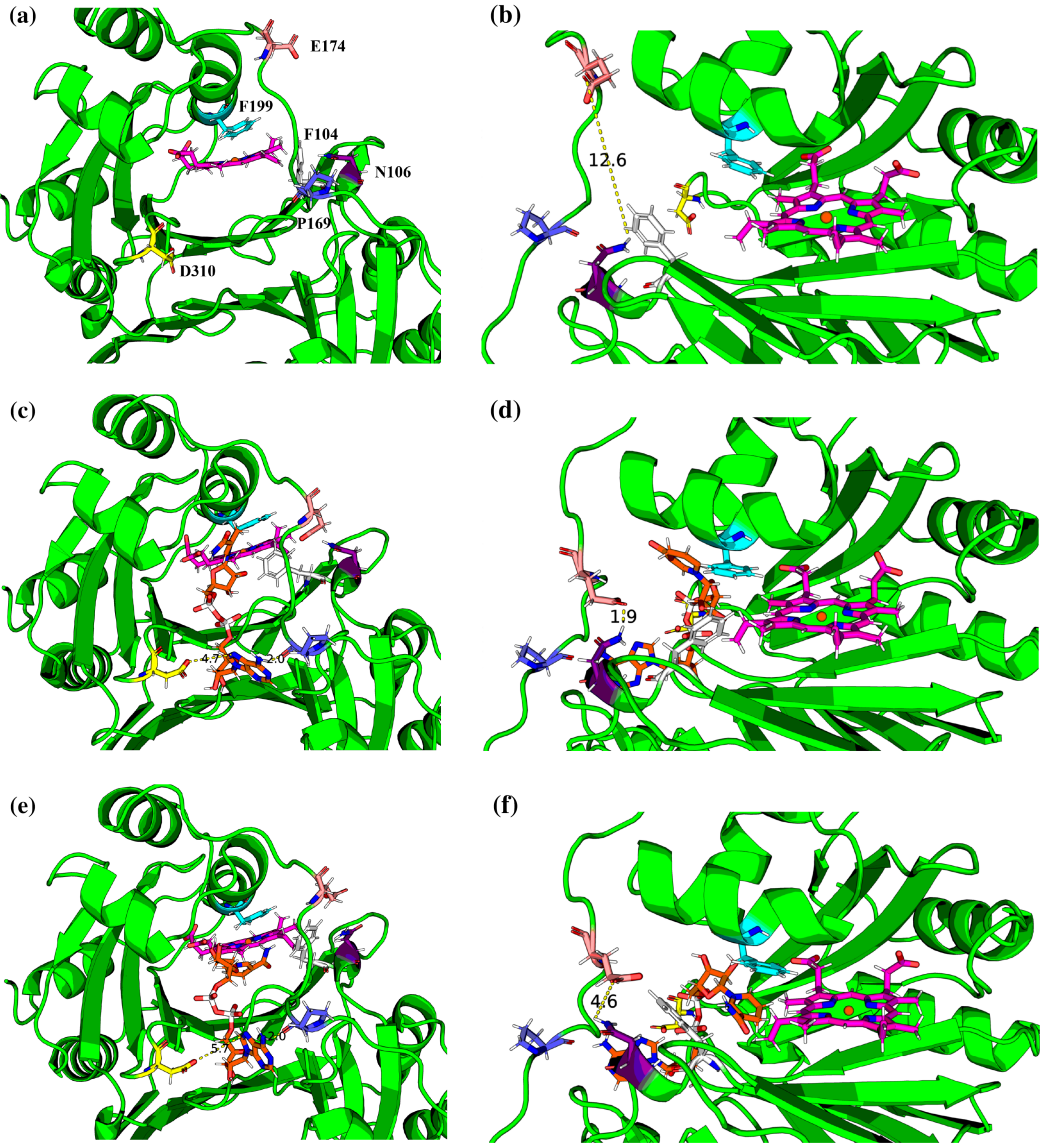
Front (left) and side (right) views illustrating the long‐range effect of NADH‐binding on F104 conformation. Haem is represented with a magenta skeleton, NADH (where present) in orange, F104 in white, N106 in purple, P169 in blue, E174 in salmon, F199 in cyan, and D310 in yellow. (a, b) Selected representative minimum when NADH is absent. (c, d) Selected representative minimum when NADH occupies the edge of the pocket. (e, f) Selected representative minimum when NADH is extended into the pocket.

This predominance of the double phe‐gate being in a closed‐opened conformation, except when NADH is near to haem, is very surprising given that simulations without NADH show a preference for the opened‐opened state. Indeed, nearly all minima had an opened‐opened double phe‐gate, with very few instances of F104 closed, as shown in Figure 3. These results suggest that F104 typically switches from an open to closed conformation upon inclusion of NADH at the pocket edge, only to then open up again as NADH unfolds and approaches haem. This behavior indicates that the double phe‐gate is indeed regulating the protein response to NADH, and thereby exerts an element of control in NADH access to haem. Further analysis of F104 flipping upon NADH inclusion was investigated. Since NADH first accesses HemS at the very edge of the main pocket, yet F104 is buried in the center, this process is a long‐range interaction mediated by a series of other residues. The full picture is provided in Figure 4.

Figure 4a,b depicts a representative minimum when NADH is absent. In this situation, the cavity is open wide, with P169 and D310 far apart. E174 is also far from N106 (12.6 Å), which frees up N106 to form a polar contact with F104, keeping the latter in an open conformation. Figure 4c,d, meanwhile, depicts a representative minimum when NADH is folded and occupies the edge of the HemS pocket. In this position, NADH prompts conformational changes to certain residues at the pocket's edge, which cascades through to the gate, causing it to shut. To be precise, P169 and D310 hydrogen‐bond to the adenine nucleobase of NADH from different sides (with 2.0 and 4.7 Å bond lengths, respectively), narrowing the cavity entrance. This movement brings E174 in close contact (1.9 Å) with N106, which itself shifts in conformation to make this bond, leaving F104 free. F104 changes from an open to closed conformation in order to form a T‐shaped *π*–*π* bond with F199. This movement blocks NADH from easy access to haem. Figure 4e,f depicts a representative minimum when NADH is extended into the pocket. In this situation, P169 and D310 still dock the adenine nucleobase of NADH in place (2.0 and 5.7 Å, respectively). However, the approach of the nicotinamide head of NADH to the double phe‐gate has caused E174 to shift back upwards. To remain bonded, N106 therefore changes its conformation considerably, ultimately yielding a bond 4.6 Å long. This movement of N106 frees up the space required for F104 to flip back to an open conformation, which in turn provides more space for NADH to slip between the two phenylalanine residues and access the haem molecule.

### 2.3 Mutating NADH‐regulating residues

F104A, F104I, F104AF199A, and F199A mutants were expressed in order to investigate the effect of partial, or full, removal of the double phe‐gate. F104I was intended to preserve the sterics of the gate and instead probe whether a change in electronic structure (specifically, the disruption of the T‐shaped *π*–*π* interaction) would affect the gate.

The mutants were studied in parallel via computations using the energy landscape approach and standard experimental methods in the laboratory, to determine whether the calculations could usefully predict and explain experimental behavior.

#### 2.3.1 Computational approach

**FIGURE 5:**
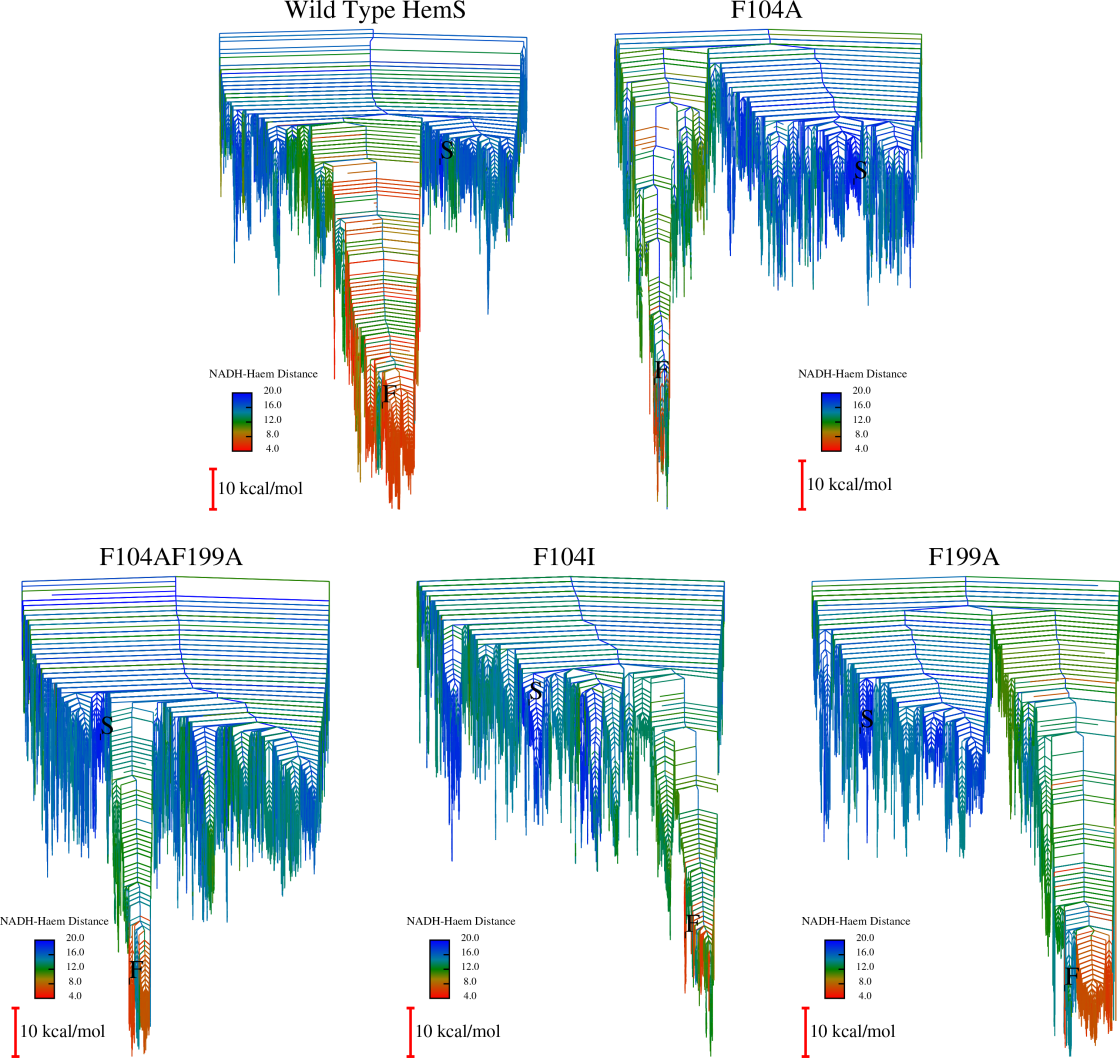
WT and mutated HemS disconnectivity graphs.

Mutations were applied, new databases generated and the results depicted as disconnectivity graphs in Figure 5, with the WT database provided for comparison.

The first, and key, property common to these disconnectivity graphs is that they all have a clearly defined funnel, leading to minima with low NADH–haem distances. These funnels suggest that all of the mutants will still be able to engage in the haem breakdown process. However, compared to the WT, all of the funnels are narrower, and fewer structures with NADH and haem in close proximity are available. These data imply that all of the mutations have had a deleterious effect and that the WT is most effective at bringing NADH and haem into close proximity and thus bringing about the reaction, which was expected given that it is the natural, evolved structure. Nevertheless, it is somewhat surprising that those mutants, where at least part of the double phe‐gate has been removed, are less effective at bringing NADH and haem together. We might have expected that NADH would be able to pass more easily through the pocket in the absence of such a gateway. It is particularly intriguing that the total removal of the double phe‐gate (F104AF199A) leads to a landscape with a narrow funnel and many low‐lying kinetic traps.

**FIGURE 6:**
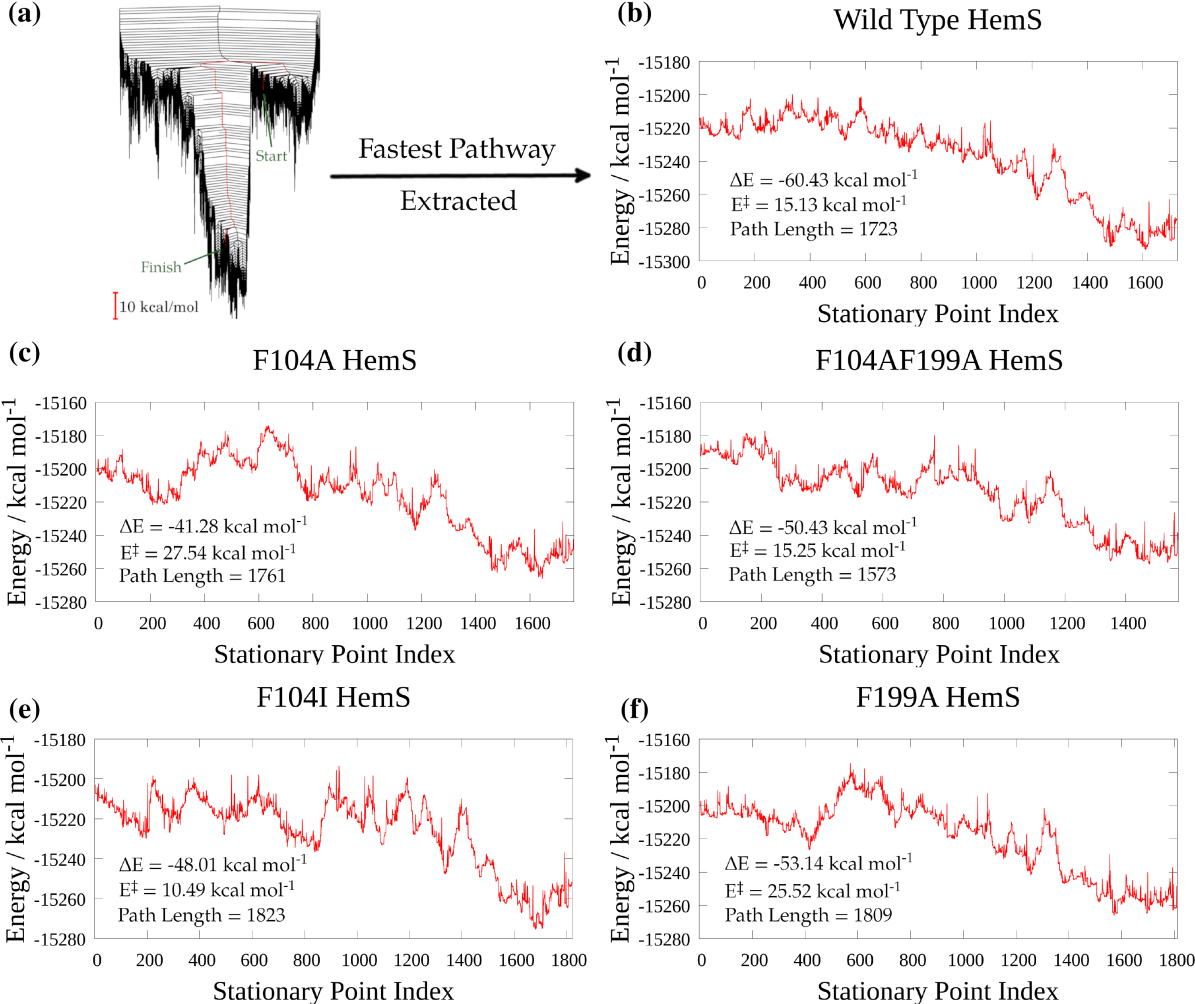
Fastest pathways, extracted using Dijkstra analysis (Dijkstra 1959; Evans and Wales 2004). (a) WT energy landscape, with the Dijkstra fastest pathway between minima S and F mapped in red. (b–f) Potential energy profiles of the fastest pathways between minima S and F, extracted from the respective energy landscapes for each mutation (see Figure 5 for these landscapes). In each pathway, the stationary point index denotes the interconnected minima and transition states connecting S (indexed as minimum 1) to F (the uppermost index). Therefore, minima are denoted as odd‐numbered indices, and transition states as even‐numbered indices. Thermodynamic changes, Δ*E*, energy differences between the highest energy transition states and the starting minima, *E* ^‡^, and path lengths are superimposed on each pathway.

Two minima, S (for start) and F (for finish), are highlighted on each graph. For the original WT system, S represents a selected minimum where NADH is folded up and at the edge of the cavity. Following mutation and reoptimization, this S minimum appears in all of the other databases, and it is highlighted in each of the mutant graphs. F, meanwhile, represents the minimum where the distance between the NADH hydride‐bearing carbon and haem *β*‐*meso*‐carbon is shortest (and therefore is taken to be a good approximation for the configuration where hydride transfer is most likely to occur). Together, S and F therefore provide good endpoints to illustrate NADH unfolding and progressing through the pocket towards haem. Using these endpoints, fastest pathways were extracted for each system using Dijkstra analysis (Dijkstra 1959; Evans and Wales 2004), as depicted in Figure 6.

In this plot, the pathways correspond to series of minima (odd numbers, starting at 1, the S minimum) and the transition states (even numbers) that connect them. Δ*E* represents the overall potential energy change from S to F. The fact that the greatest energy stabilization (Δ*E* = −60.43 kcal mol^−1^) occurs in the WT case provides further evidence that all of the mutations are deleterious. It is interesting that the F104AF199A mutant has the shortest integrated path length, suggesting that entire removal of the double phe‐gate allows NADH to move through the cavity and approach haem more directly. This new pathway, however, introduces an extended barrier not found in the WT, corresponding to the section of the pathway between minima 685 and 955. This region of the pathway corresponds to an adjustment of the nicotinamide head in the pocket, a process that in the WT involves interaction with both F104 and F199, and so these two residues therefore provide a stabilizing effect.

Given that the fastest pathway for F104AF199A effectively has the same *E* ^**‡**^ value as the WT, and the path length is significantly shorter it would be, at face value, unexpected were the WT to have a faster rate of reaction than this double mutant. Indeed, that is exactly what was seen experimentally, as shall be detailed in section 2.3.2. However, upon closer inspection, the energy landscapes approach provides evidence for this conclusion, which is difficult to replicate with other computational methods. First, as described above, the comparison of the Dijkstra fastest pathways for the WT and F104AF199A shows that the double mutation introduces a trough and extended barrier, that is, the path becomes rougher and, therefore, more energy is required to traverse it. Second, the energy landscapes for the WT and F104AF199A (see Figure 5) are notably different. Whereas the WT energy landscape has a wide funnel with multiple minima where NADH and haem are in close proximity, such minima are fewer in the F104AF199A landscape, and the funnel much narrower. This situation suggests that removal of the double phe‐gate creates a landscape where there are fewer possible low‐energy configurations with the ligands in close proximity. Furthermore, the F104AF199A landscape has many more low‐lying minima separated by large barriers from the main funnel than the WT landscape has. These low‐lying minima constitute kinetic traps. Though avoided by the Dijkstra “fastest” pathway, these traps play a significant role in governing the overall kinetics of biological systems (Dutta and Pollak 2021; Joseph et al. 2017; Swinburne et al. 2020; Wales 2022). NADH, when entering the pocket, may follow a pathway which falls into one or many of these traps, thus hindering its overall progress towards haem.

**FIGURE 7:**
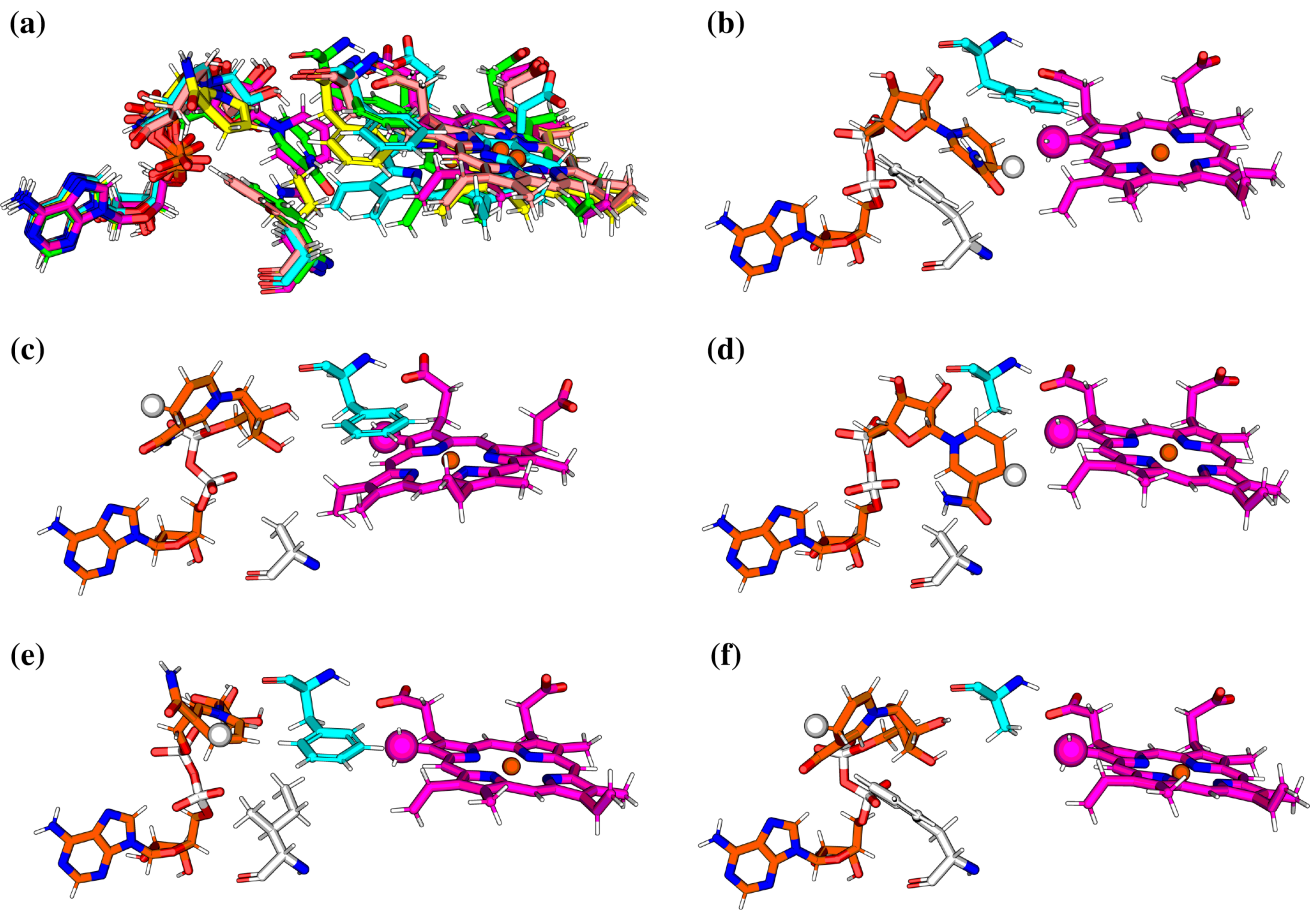
Lowest energy structures for WT and mutated HemS with haem and NADH, represented in a reduced form. Only haem, NADH and residues 104 and 199 of HemS are depicted. (a) Overlay of all structures shown in (b–f), where the WT system is depicted in green, F104A HemS in cyan, F104AF199A in magenta, F104I in yellow, and F199A in pink. (b–f) Reduced representations depicting haem (magenta), NADH (orange), and residues 104 (white) and 199 (cyan) of HemS. The (*R*)‐hydride of NADH and methyl C5 atom of haem are highlighted as enlarged spheres. (b) WT, C5‐hydride distance: 2.9 Å. (c) F104A, C5‐hydride distance: 12.3 Å. (d) F104AF199A, C5‐hydride distance: 3.4 Å. (e) F104I, C5‐hydride distance: 9.2 Å. (f) F199A, C5‐hydride distance: 12.9 Å.

Similarly, the F104I mutation has a lower *E* ^‡^ value than the WT which, at face value, would potentially suggest that this mutation will bring NADH and haem together at a faster rate. However, a comparison of the landscapes reveals that there are fewer low‐energy configurations where NADH and haem are in close proximity for F104I, and its landscape has many more low‐lying kinetic traps. Such features, along with a comparison of the lowest energy structures (see Figure 7), provide a detailed explanation for the results discussed in section 2.3.2.

The lowest energy minima from each landscape were also investigated. These structures are given in Figure 7. Interestingly, all of the mutations involving the double phe‐gate had a significant impact on the properties of the lowest energy minimum. For the WT case, the lowest energy structure has atoms C5 (a methyl carbon) of haem and the (*R*)‐hydride of NADH within 2.9 Å of each other, with the hydride pointing directly at this methyl carbon. This finding constitutes the strongest evidence concerning which haem carbon is attacked, given the absence of any definitive experimentally derived data. Should the reaction proceed via this methyl carbon, subsequent electronic rearrangement will be required in order to cleave the porphyrin ring at the *β*‐meso‐carbon position, which experiment has confirmed does occur (see our previous report (Keith et al. 2023) for details).

For all the energy landscapes analyzed, when the double phe‐gate is disrupted, the lowest energy structure no longer has the hydride‐bearing carbon of NADH in close proximity and presenting one of its hydrides to the porphyrin, primed to react. Instead, the nicotinamide head points away from haem or, in the case of F104AF199A, is oriented so that both hydrides are situated perpendicular to haem instead. Typically, given evolutionary pressures, the lowest energy structure within an enzymatic landscape will be optimal, or very near to optimal, for facilitating its associated reaction. For some systems, a very small number of mutations may improve enzymatic activity. A greater number will show little effect, and yet others may disrupt the overall stability of the protein while leaving the lowest energy structure essentially the same. Given that all of these mutations to the double phe‐gate have resulted in significant changes to the lowest energy structures, this result suggests that these mutants do not drive a hydride of NADH towards haem so effectively as the wild type does, thus reducing the efficacy of the breakdown process.

#### 2.3.2 Experimental approach

**FIGURE 8:**
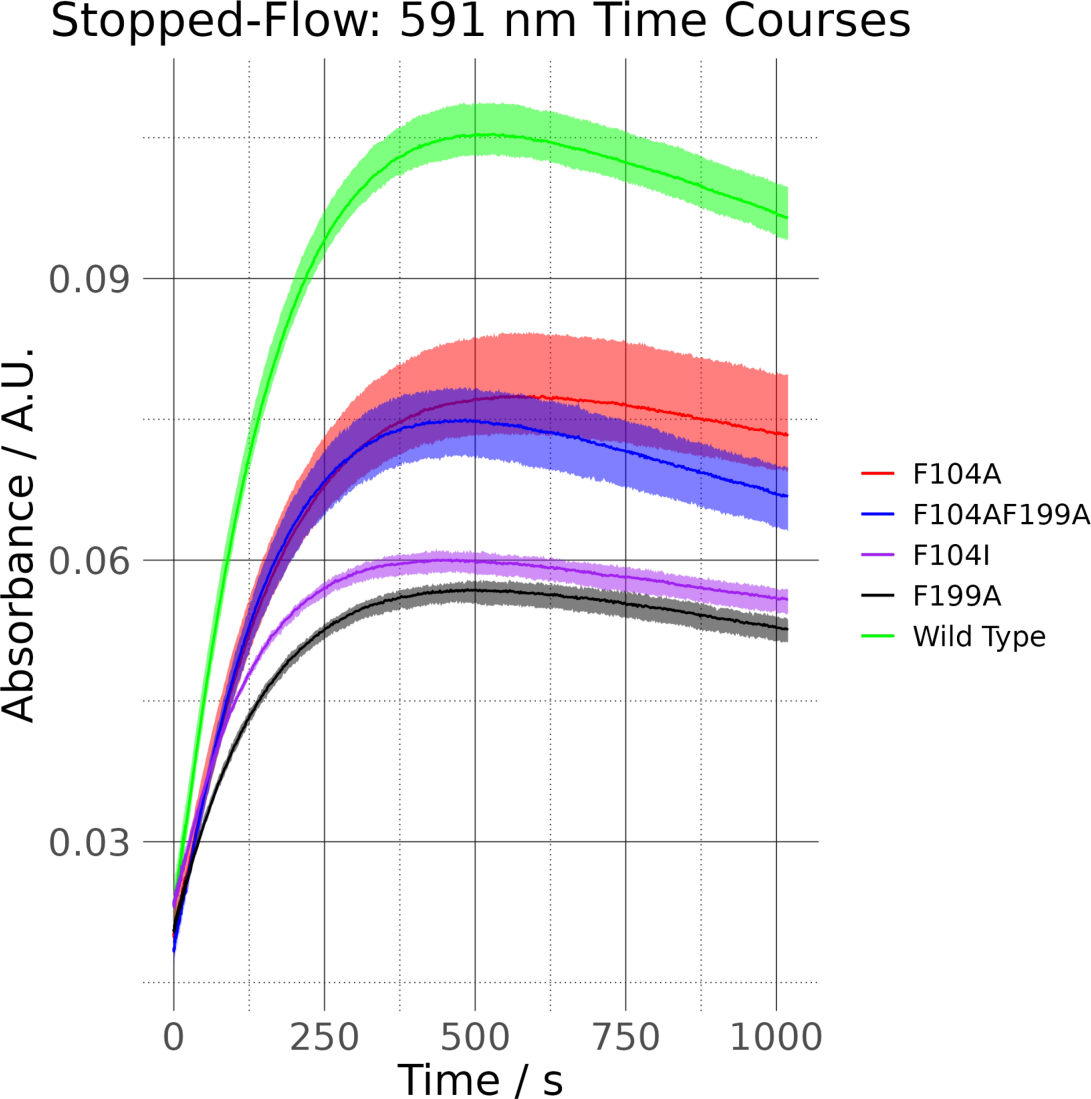
Stopped‐flow spectroscopy time courses, tracking peak evolution at 591 nm over 1000 s. Assays were done in triplicate. Solid lines chart the average absorbance values, with the shaded envelopes encompassing the maximum and minimum values at each time point.

UV–visible spectroscopy confirmed that the mutants retained haem‐binding properties similar to those observed for the wild type (see Figure S4 and Table S3). Further analysis also showed that NADH‐dependent haem breakdown was retained (see Figure S5), in line with our computational predictions. However, this technique is too low resolution to determine whether the mutations influence the rate of breakdown. Therefore, further experiments were conducted using stopped‐flow spectroscopy, where time courses measuring the change of absorbance at 591 nm, the signature peak of the haem‐breakdown product, were recorded. The results are given in Figure 8 and Table 1.

**TABLE 1:** Stopped‐flow spectroscopy data.

	*A*_0.0391 s_	*A*_99.515 s_	*t*_peak_	*A*_peak_	*R*_100 s_/μs^−1^	*R*_peak_/μs^−1^
WT HemS	0.0232 (29)	0.0635 (24)	529.433	0.1054 (30)	404 (67)	155 (24)
F104A	0.0199 (27)	0.0470 (32)	571.425	0.0775 (59)	273 (55)	101 (21)
F104AF199A	0.0185 (16)	0.0477 (21)	483.442	0.0750 (36)	293 (38)	117 (16)
F104I	0.0238 (11)	0.0446 (6)	459.446	0.0600 (11)	209 (13)	79 (5)
F199A	0.0206 (9)	0.0401 (7)	487.441	0.0569 (13)	196 (13)	75 (5)

*Note*: Peak evolution at 591 nm was tracked for the WT plus the F104A, F104AF199A, F104I, and F199A mutants over a 1000 s interval. *t*_peak_ is the time at which the absorbance reaches a maximum value, *A*_peak_. *R*_100 s_ is the rate of reaction over the first 100 s, calculated between times 0.0391 s and 99.515 s. *R*_peak_ is the rate of reaction from the first time point (*t* = 0.0391 s) to *t*_peak_. Assays were run in triplicate, and bracketed values are standard deviations.

The time courses in Figure 8 show that the initial rate of haem‐breakdown is markedly reduced for all of the mutations with respect to the wild type. Also, less of this initial breakdown species is produced before the rate of its loss exceeds its rate of formation, as indicated by the downturn of the curves. Table 1 gives numerical details, showing that the initial rates of reaction (over the first 100 s) and the rates of reaction up to the maximum concentration of the 591 nm species are significantly reduced upon phe‐gate mutation. Such findings are in line with our computational predictions that, despite the removal of bulky residues between the two main ligands, the rate of reaction will be reduced rather than improved. It further aligns with our predictions that conversion of both of the gates to alanine will have a less significant effect on the rate, than conversion of just one or the other. The F104I data also show that, despite conversion to a residue of similar size, the rate of reaction is reduced, suggesting that the double gate is not merely physical but has an electronic component too. These data align with the lowest energy structure predicted in Figure 7, where the different electronic environment “pushes” the NADH nicotinamide head away from the haem molecule.

### 2.4 Crystallography

**FIGURE 9:**
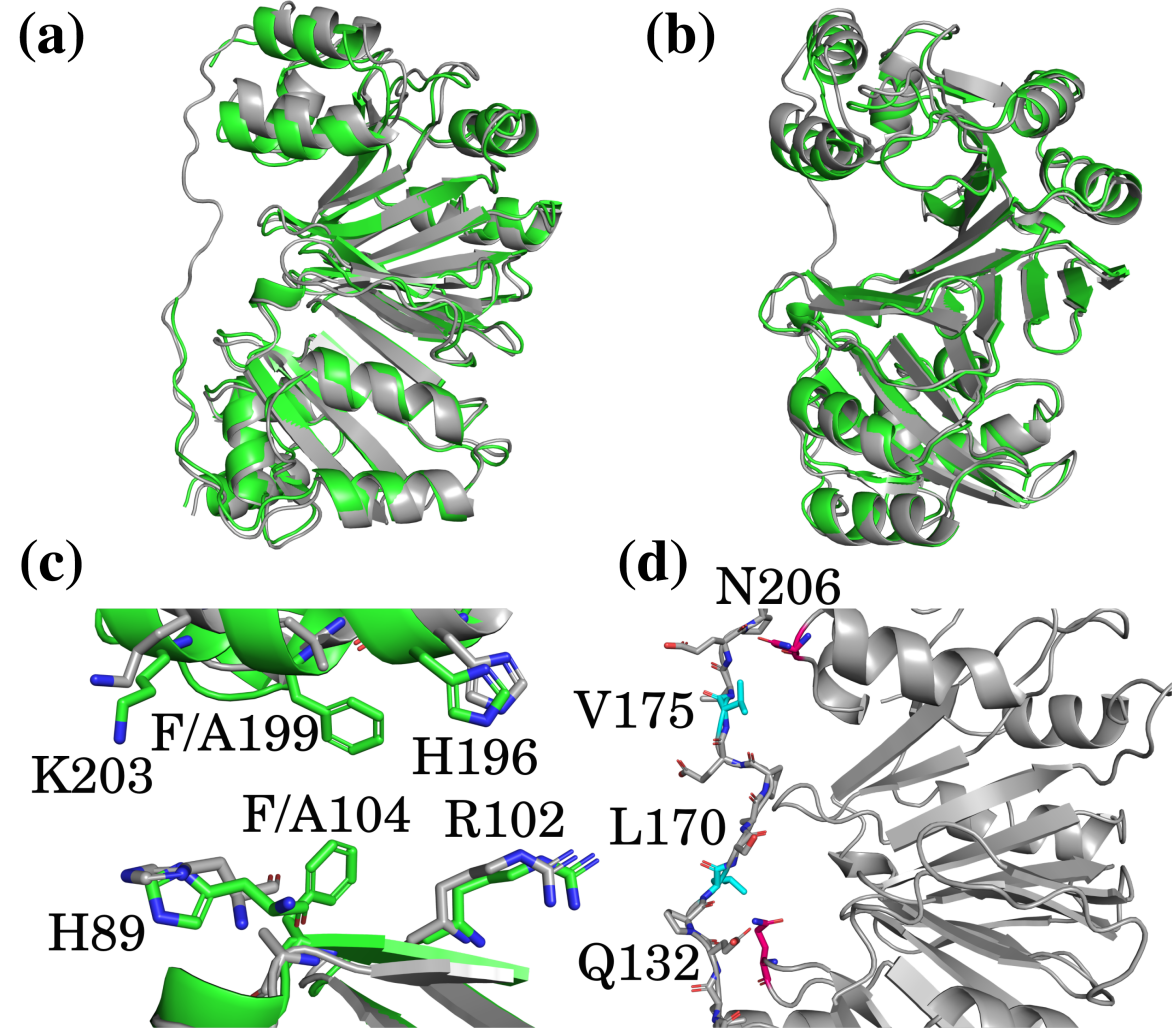
Crystal structures of WT (green) and F104AF199A (gray, PDB: 7QXV) HemS. (a, b) Overlaid structures, from two different perspectives. The WT unstructured loop is broken due to poor resolution of these connecting residues but the F104AF199A loop is complete. (c) Magnification of the main pocket, with key residues highlighted. (d) Magnification of the complete F104AF199A HemS extended loop. Key hydrophobic residues within this loop are highlighted in cyan, and the residues they engage in stabilizing interactions with are highlighted in magenta.

Crystals were grown and analyzed for WT and F104AF199A HemS, as described in the methods. The resolved structures showed significant rearrangement of the C‐terminal domain. The mutation of the double phe‐gate at the center of the main cavity caused significant structural changes. Figure 9a,b shows these structures superimposed, suggesting that removal of the gate opens up the main pocket significantly, with the capping *α*‐helix becoming less deeply buried. We were surprised by this result as we had assumed that the conversion of bulky to small residues within the main cavity would result in the protein rearranging to reduce the cavity volume. We noticed that the conformation of Q200 changes upon the double mutation so that it no longer points away from the cavity, instead pointing parallel to the helix, and we hypothesize that the helix moves upwards to compensate for the space now left immediately above it. Figure 9c gives a magnified representation of the main pocket, and shows how removal of the double phe‐gate results in the rearrangement of key residues within the pocket. The residues which axially bind haem, H196 and R102 (via OH^−^ or H_2_O) are compressed together by 0.4 Å, which will affect the extent of structural distortion these residues impose on haem. Furthermore, loss of the gate causes the orientations of both H89 and K203 to change. The change to K203 in particular will affect the method of NADH entry and passage through the pocket, given its role in interacting with the nicotinamide head of NADH as this ligand unfolds.

In the WT case, the unstructured loop connecting the N‐ and C‐terminal domains was not fully resolved, as in previous literature (Schneider et al. 2006; Schneider and Paoli 2005). However, in the F104AF199A case, resolution proved to be higher, and this “missing loop” could be constructed. It may be the case that the widened cavity caused by the double mutant stretches the loop so that its structural flexibility is reduced, thereby making resolution easier. Nevertheless, as far as the authors are aware, this structure is the first within this family of haemoproteins in which the monomeric structure has been fully resolved. Since this unstructured loop caps the region of the large pocket where NADH enters, the full resolution may provide insight into any potential role it plays in protein–ligand association. Also, close inspection of the loop shows how other sections of the protein interact with hydrophobic residues within the loop to confer stability. Despite the loop being exposed at the edge of the protein, L170 and V175 are oriented inwards, where they are proximate to Q132 and N206, respectively. The amide groups of asparagine and glutamine are both able to stabilize hydrophobic amino acids by creating “hydrophobic pockets” through hydrogen bonds with surrounding water molecules (Selvaraj et al. 2023), an effect which we propose is occurring here.

## 3 CONCLUSIONS

HemS belongs to a wider family of haemoproteins about which little is known. The discovery that HemS and related homologues can anaerobically break down haem could explain how certain pathogens can survive in oxygen‐deficient regions of host organisms. Since the source of HemS is *Yersinia enterocolitica*, which is implicated in yersiniosis, plus one close homologue is ShuS, from *Shigella dysenteriae*, the bacterium primarily responsible for dysentery, a detailed understanding of this chemical process could have significant implications for human health.

Computational analysis of the WT pathway highlighted the importance of residues F104 and F199, which together comprise a double phe‐gate. This feature changes conformation as NADH approaches haem within the pocket. Analysis of the energy landscape for HemS and haem without NADH present showed that the majority of its minima had both components of the gate open. In comparison, inclusion of NADH causes F104 to close, unless NADH is proximate to haem. Taken together, these results suggest that the double phe‐gate regulates NADH access to haem via a sophisticated set of interactions which extend outwards to the pocket edge, thus causing it to exercise tight control over the orientation which NADH is in before the hydride transfer event.

Following analysis of the WT energy landscape, further landscapes for the selected mutants were produced. All of these landscapes showed narrower funnels, with fewer minima where NADH and haem are proximate, and more low‐lying kinetic traps than the WT case, suggesting that changes to the double phe‐gate will reduce haem breakdown efficiency. Dijkstra fastest pathways were extracted from these landscapes and inspected, which suggested that all mutations resulted in rougher pathways and/or less favorable thermodynamics for NADH unfolding and approach to haem. Overall trends between computation and stopped‐flow experiments are consistent, with particular experimental features explained from pathway visualization and database analysis.

X‐ray crystallography provided a fully resolved structure for F104AF199A HemS. The complete loop showed the hydrophobic residues, L170 and V175, in close proximity to Q132 and N206, two residues capable of forming “hydrophobic pockets” via their amide groups, indicating a method of stabilization for these two hydrophobic residues on the exposed loop. Comparison of this F104AF199A structure with the WT structure showed that removal of the double phe‐gate causes the pocket to widen and the orientation of key residues to change.

Energy landscape theory has demonstrated utility for solving difficult protein‐folding problems. It is our hope that this report has demonstrated that this approach can be extended to investigate certain problematic protein‐ligand interactions. In particular, we would recommend this approach be used to investigate cases where knowledge of precise local dynamics is important, and more conventional methods such as laboratory‐based biophysical methods or standard molecular dynamics simulations cannot be used. Many enzymes utilize gating mechanisms to regulate their activities, and we propose that the energy landscapes approach be used to explore such features.

## 4 MATERIALS AND METHODS

### 4.1 General materials and conditions

All chemicals were purchased from Merck UK unless otherwise stated. Bovine haemin stocks were from Fluka Biochemika. All solutions were purified to a resistance of 18.2 MΩ cm^−1^ (PURELAB Chorus, Veolia Water Technologies). Solution pH adjustments were made using 5 M NaOH and 5 M HCl. An InLab Micro pH probe (Mettler Toledo) connected to a PHM240 digital pH meter (Radiometer Analytics) was used to measure solution pHs, following three‐point calibration with standard IUPAC buffers. pH‐adjusted solutions were filtered with 0.2 μm membranes (Sartorius Stedim Biotech). UV–visible spectroscopy using a Cary 60 spectrophotometer (Agilent Technologies) and application of the experimental Beer–Lambert law was used to determine protein concentrations. ExPASY ProtParam (Gasteiger et al. 2005) was used to theoretically predict the $\epsilon_{280}$ value for *apo*‐HemS. To prepare haem stocks, solid hemin chloride was dissolved in 1.0 M NaOH followed by dilution with buffer solution to 1.0 mM or 0.1 mM final concentration.

### 4.2 Buffers

Affinity Chromatography Buffer A: 20 mM bis‐tris propane (BTP), 10 mM imidazole (Acros Organics), 300 mM KCl (Fisher), pH 6.5. Affinity Chromatography Buffer B: 20 mM BTP, 500 mM imidazole, 300 mM KCl, pH 6.5. Size Exclusion Chromatography Buffer (SEC): 20 mM BTP, pH 6.5. Anion Exchange Chromatography Buffer A (AEC A): Same as SEC. Anion Exchange Chromatography Buffer B (AEC B): 20 mM BTP, 500 mM KCl, pH 6.5. Thrombin His‐Tag Cleavage Buffer (THTC): 20 mM BTP, 150 mM NaCl (Fisher), 1.5 mM CaCl_2_ (VWR International), pH 8.4. Crystallization Buffer A: 50 mM HEPES (Sigma‐Aldrich), 150 mM NaCl, pH 8.0. Crystallization Buffer B: 100 mM Tris–HCl (USB Corporation), 1.8M (NH_4_)_2_SO_4_ (Breckland Scientific Supplies), 2% (w/v) PEG 400, pH 8.5.

### 4.3 WT plasmid preparation

The *hemS* gene in a pGAT2 expression vector (Peränen et al. 1996) was provided by Schneider and Paoli (University of Nottingham, UK). This sequence led to protein expression with one point of differentiation from that found in the literature (2J0P in the Protein Data Bank) (Schneider and Paoli 2005), which has a glutamic acid at residue 333, whereas the present sequence has an aspartic acid. The full sequence is provided in Section D of the SI. HemS was expressed with a His_6_‐tag connected through a thrombin cleavage site to the N‐terminal. The pGAT2 vector confers ampicillin resistance.

### 4.4 Site‐directed mutagenesis

Mutants were created from site‐directed mutagenesis on the WT gene. The four mutants made were F104A, F104AF199A, F104I, and F199A. Primers (given in Section C of the SI) were designed using SnapGene (SnapGene 2021) and ordered from Merck UK Ltd. Mutagenesis was carried out using a QuikChange II XL Kit (Stratagene, Agilent Technologies). Transformation was also carried out as suggested in the kit (heat shock of ultracompetent *E. coli* XL10‐Gold cells or competent *E. coli* XL1‐Blue cells), culminating in the transformed cells being incubated overnight on lysogeny broth (LB) agar ampicillin plates at 37°C. Successfully grown cells were selected, and mutant plasmid extracted using a Miniprep Kit (Qiagen). Purity was determined using a Nanodrop 2000C Spectrophotometer (Thermo Scientific), and the sequences confirmed by standard Sanger Sequencing (DNA Sequencing Facility, Department of Biochemistry, University of Cambridge) of the plasmids using standard T7 primers. Mutagenesis for the F199A mutant was unsuccessful using the QuikChange II XL Kit, and so the QuikChange Lightning Kit (Stratagene, Agilent Technologies) was used instead. To make the double mutant, F104AF199A, point‐mutations were done successively.

### 4.5 Protein expression and purification

HemS expression and purification was as described in our previous report (Keith et al. 2023). Briefly, expression used electrocompetent *E. coli* BL21‐Gold (DE3) cells (Agilent Technologies), and transformation was by electroporation. Cell lysis used a high pressure homogenizer (EmulsiFlex‐C5, Biopharma Process Systems). The first round of purification was achieved using affinity chromatography with high density Ni resin. Thrombin was then added to remove the His‐Tag, and affinity chromatography used again to separate cut from uncut protein. Size exclusion chromatography provided a final round of protein purification. Purified protein was kept in 20 mM bis‐tris propane (BTP), pH 6.5 buffer solution. Yield of protein was typically 20 mg per 1 L *E. coli* cells harvested. An SDS gel is provided in Figure S2.

### 4.6 UV–visible spectroscopy

Data collection was either on a Cary 60 (Agilent Technologies), Cary 100 (Varian Ltd.), or Cary 400 (Varian Ltd.) spectrophotometer. Standard 9/9/B quartz cuvettes with 10 mm pathlength (Starna Scientific) were used throughout. A scan rate of 300 nm min^−1^ was typically employed.

#### 4.6.1 Haem‐binding–WT, F104AF199A, F199A assays

These haem‐binding experiments followed the method described in our associated report (Keith et al. 2023). Briefly, a baseline of 20 mM, 100 mM KCl, pH 6.5/8.0 buffer was taken, and protein then added to approximately 10 μM. Calibrated haem was then added to exactly 5 μM, and scans taken until the spectra stabilized.

#### 4.6.2 *Steady state reaction of* holo*‐HemS with NADH*

Unless otherwise stated, the baseline consisted of the protein, haem and buffer, and data collection started upon injection of NADH. After multiple experiments, it was recognized that thorough pre‐mixing of protein and haem was essential to allow haem to fully bind before recording the baseline. All experiments were maintained at 25°C by a Peltier heating block, and spectra were recorded in 1 nm increments using a spectral bandwidth of 1 nm with full slit height.

### 4.7 Mass spectrometry

Liquid chromatography mass spectrometry (LCMS) data collection used a Xevo G2‐S ToF mass spectrometer (capillary voltage 2.0 kV, cone voltage 40 kV; desolvation temperature 350°C) coupled to an acquity UPLC BEH300 C4 column (1.7 μm, 2.2 × 50 mm). H_2_O with 0.1% formic acid (Solvent A) and 95%:5% acetonitrile:H_2_O with 0.1% formic acid (Solvent B) formed the mobile phase. The flow rate was specified as 0.2 mL min^−1^. The gradient was run according to the routine: 95% A for 0.93 min; gradient to 100% B for 4.28 min; 100% B for 1.04 min; and gradient to 95% A for 1.04 min. The desolvation gas used was nitrogen, which was set to a total flow of 850 L h^−1^. The maximum entropy (MaxEnt) algorithm (Merow et al. 2013) pre‐installed on the MassLynx software (Waters, v4.1) was used to read the ion series in order to reconstruct total mass spectra. Deconvoluted spectra were split into 0.25 Da channels, and applied a width at half‐peak height of 0.75 Da. Masses are provided in Table S1.

### 4.8 X‐ray crystallography

#### 4.8.1 Crystallization

Pre‐mixed 10 μM protein: 10 μM heme were reacted with 2 mM NADH anaerobically in a total volume of 17.6 mL. The product mixture was exchanged into Crystallization Buffer A and concentrated to 30 mg mL^−1^ using centrifugation (5700 rpm, 4°C) with 10 kDa Amicon Ultra centrifugal filters. Five microliter samples were pipetted onto individual glass coverslips before being mixed with 5 μL Crystallization Buffer B. These coverslips were then placed above individual wells for crystallization by the hanging drop method, with 700 μL Crystallization Buffer B added to each well to act as the precipitant solution. Crystals were incubated at 277 K for 2–4 weeks.

#### 4.8.2 Data collection and analysis

Cryoprotection of the crystals was conducted using a solution of 0.1 M Tris–HCl pH 8.5, 1.8 M ammonium sulfate, 2% PEG 400, and 1.2 M sodium malonate. Crystals were cryo‐cooled in liquid nitrogen for data collection. X‐ray diffraction data were collected at the Diamond Light Source (Didcot, UK) and data derived from automated data processing using autoProc (Vonrhein et al. 2011) were utilized for the structure determination. Structures were solved by using programs in the CCP4 package (Winn et al. 2011). Models were iteratively refined and rebuilt using the Refmac (Murshudov et al. 1997) and Coot (Emsley et al. 2010) programs. The crystal structure for F104AF199A HemS is published in the Protein Data Bank with ID 7QXV. Crystallographic data is provided in Table S4 and Figure S6.

### 4.9 Stopped‐flow spectroscopy

All experiments were run on an SX 20 stopped‐flow spectrometer (Applied Photophysics). The light source was provided by a Xe lamp, and entrance/exit monochromator slit widths were both set to 2 mm. A photodiode array (PDA) with resolution 1.2 nm and a range of 250.0–722.9 nm was used. The step size between wavelengths was set to 2.2 nm. The integration period was set to 1.260 ms, scan period to 1.097 ms, offset to 0 V, and gain to 127. A water bath kept the temperature at 25°C. All spectra were taken against a baseline of 20 mM BTP, pH 6.5. Unless stated otherwise, experiments consisted of a 1:1 pre‐incubated mixture of protein and haem being mixed with excess NADH. Typically, 2000 time points were recorded over 250 s. Data analysis included the use of ProK‐IV software (Applied Photophysics).

### 4.10 Relibase^+^

The (now retired) Relibase^+^ bioinformatics package (Hendlich 1998) was run according to the developers' instructions (Schmitt et al. 2002). The settings detailed extensively in Choy (Choy 2015) were followed. Briefly, Relibase^+^ used CavBase (Schmitt et al. 2001; Schmitt et al. 2002), a program that generalizes cavities according to the degree of burial and the hydrogen‐bonding and aromatic properties of its flanking residues, thus generating “pseudocentres.” CavBase, in turn, uses LIGSITE (Hendlich et al. 1997), a program which defines cavities by placing the protein of interest inside a 3D spatial lattice, and scoring the protein atoms according to the overlap of their van der Waals radii with the lattice grid points. Relibase^+^ then compared these pseudocentres against the cavities of other proteins, with the Protein Data Bank used as a database. When applied to HemS, LIGSITE identified the haem‐binding site as being one of two sub‐pockets within a larger cavity. Using residues F104 and F199 as the dividing line between the two sub‐pockets, CavBase generalized the non‐haem‐binding sub‐pocket, and comparison against other proteins was achieved using Relibase^+^. This analysis gave a 21.1% (regarded as weak) hit to PDB structure 2AQI, a mutant of the 2‐trans enoyl‐acyl carrier protein reductase enzyme InhA in complex with NADH (Oliveira et al. 2006). When superimposed onto the HemS structure, this NADH molecule produced only minor atomic clashes, with its nicotinamide head stretched towards the *β*‐*meso* carbon of haem, seemingly in a prime position to transfer a hydride.

### 4.11 GPU implementation

The AMBER interface to the Cambridge energy landscape software was employed (Mantell et al. 2016).

### 4.12 Starting configurations and force field settings

These settings followed those described in our previous report (Keith et al. 2023). The published *holo*‐HemS structure (PDB: 2J0P) (Schneider and Paoli 2005) provided initial coordinates. Solvent and crystallant molecules were removed, and Swiss‐PdbViewer (Guex and Peitsch 1997) used to reconstruct the missing loop. Haem parameters were taken from those implemented by Giammona (Giammona 1984). For those systems requiring NADH, this molecule was added using the coordinates determined by Relibase^+^ (Hendlich 1998) and using the parameters implemented by Walker (Pavelites et al. 1997; Walker et al. 2002). Following benchmarking exercises, all simulations were run using the ff99SB force field (Hornak et al. 2006) from the AMBER12 package (Case et al. 2012), with the implicit generalized Born, *igb2* (Onufriev et al. 2000), solvent model (no cutoff, and with effective monovalent ionic concentration of 0.1M using the Debye‐Hückel approximation) (Srinivasan et al. 1999).

### 4.13 Landscape exploration: System sampling

Basin‐hopping global optimization (Li and Scheraga 1987; Li and Scheraga 1988; Wales and Doye 1997), as implemented in the gmin program (Wales 2021a) interfaced with AMBER12, was used to grow the *holo*‐HemS (with and without NADH) databases, and to identify low‐lying minima.

### 4.14 Landscape exploration: Creating kinetic transition networks

Kinetic transition networks (KTNs) were generated using the discrete path sampling (DPS) strategy (Wales 2002; Wales 2003; Wales 2004), as implemented in the optim program (Wales 2021b) interfaced to AMBER12. Transition state candidates were obtained using the doubly‐nudged elastic band (DNEB) algorithm (Trygubenko and Wales 2004a; Trygubenko and Wales 2004b), which were subsequently converged using hybrid eigenvector‐following (HEF) (Cerjan and Miller 1981; Munro and Wales 1999; Pancíř 1975). The local minima immediately connected to these transition states were then discerned using an L‐BFGS‐derived steepest‐descent algorithm (Liu and Nocedal 1989; Nocedal and Wright 2006). Picked minima were then connected to one another iteratively using the Dijkstra shortest path algorithm (Dijkstra 1959), which describes the total set of minima as a weighted, directed graph (Carr et al. 2005). This method allows for the assembly of a priority list to efficiently attempt connections based on edge weights. To further increase connectivity and refine the KTN, the pathsample program (Wales 2021c) (which acts as a driver for optim) was used. The pathsample strategies employed were the shortcut scheme (Carr et al. 2005; Joseph et al. 2017; Strodel et al. 2007), which shortens artificially long pathways; the shortcut barrier scheme (Joseph et al. 2017; Strodel et al. 2007), which removes artificially large barriers; the untrap scheme (Joseph et al. 2017; Strodel et al. 2007), which removes artificial kinetic traps; and the connectunc scheme (Röder and Wales 2018), which connects unconnected minima to the main set, until rate constants are converged. A new feature of the connectunc scheme was developed specifically for Keith et al (Keith et al. 2023) and the present work. By considering all of the sets of interconnected stationary points (known as sub‐databases) within an overall database, the new algorithm determines the fewest and narrowest conformational gaps that must be filled in order to connect all sub‐databases (and, therefore, by implication, all stationary points) within the database. This calculation is achieved through a consideration of which sub‐databases would be optimal to attempt to connect directly, as well as which minima within those sub‐databases to select for connection. This approach allowed for the efficient connection of clusters of stationary points with NADH at various points of progression along the HemS pocket. For further details, see Keith (2021) and Keith et al. (2023).

### 4.15 Templating strategy

To generate relevant starting coordinate and topology files for the mutants under consideration, a strategy using the WT system as a “template” was used, giving an estimated 10–20 fold speed‐up. See our previous report (Keith et al. 2023) for details.

### 4.16 Visualization and analysis of landscapes

Visualization of structures and pathways was achieved using PyMol (Schrödinger and DeLano 2020) and VMD (Humphrey et al. 1996).

### 4.17 Disconnectivity graphs

**FIGURE 10:**
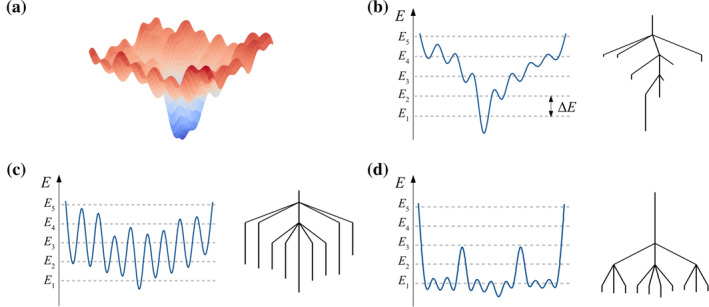
Methods of representing energy landscapes. (a) Standard, textbook representation of a potential energy surface. This type of representation is commonly insufficient for encapsulating the important features of a complicated biomolecular system with many degrees of freedom. (b–d) Pictorial correspondence between standard 2D‐representations of a potential energy surface and disconnectivity graphs for three different, simple energy landscape types (Wales 2003). *E* represents the system energy, and *E*_*n*_ to energies at which a superbasin analysis was conducted. (b) “Palm tree” motif. (c) “Weeping willow” motif. (d) “Banyan tree” motif.

Large biomolecular systems contain numerous degrees of freedom and therefore the number of possible minima and transition states, which scale exponentially with system size (Stillinger and Weber 1982), is vast. Representing such data is therefore difficult. Textbook‐style depictions of potential energy surfaces, as in Figure 10a, are unsuitable as these only convey 3D representations of the landscapes, when in reality the dimensionality is significantly higher. Since high‐dimensional objects are impossible for individuals to visualize, an alternative method for representing these stationary points faithfully is therefore required.

Therefore, disconnectivity graphs (Becker and Karplus 1997; Wales 2003; Wales et al. 1998) were used to visualize the energy landscapes. The line endpoints in these graphs correspond to minima, and nodes define superbasins. The vertical axis represents the energy of the system, whereas the horizontal axis is a free variable which can be tuned by the user to give the clearest representation of the data. Minima can be classified into disjoint sets, known as superbasins (Becker and Karplus 1997), by running analyses at fixed energy intervals, *E*_*n*_. Minima occupy the same set (or superbasin) if a complete discrete path, containing intermediate minima and transition states, connects these minima without the energy, $E_{n}$, being exceeded. Superbasins are therefore marked on the graph by nodes at these corresponding energies, $E_{n}$. Minima located in separate superbasins need to traverse the appropriate node(s) in order to interchange (i.e., such a situation occurs when one or more stationary points along the pathway separating the minima of interest has an energy above $E_{n}$). Various structural features of the energy landscape then become apparent, providing a useful pictorial guide. This construction is illustrated in Figure 10b–d. A useful analogy for disconnectivity graphs are “trees,” which contain various “branches.” Disconnectivity graphs displaying a “palm tree” motif, as illustrated by the example in Figure 10b, are indicative of landscapes containing a steep funnel with low barriers. Biomolecular landscapes typically have this motif, as evolution customarily drives an optimization of energetics so that a well‐defined structure can easily be achieved. Alternatively, disconnectivity graphs with a “weeping willow” motif (Figure 10c) arise when the landscape has a shallow funnel with large barriers, and disconnectivity graphs with a “banyan tree” motif (Figure 10d) arise when the landscape is “rough” and contains many competing low‐energy minima, a feature which is more typical of glassy rather than biomolecular systems.

Graphs can be color‐coded to highlight further properties of the system. In the present work, color‐coding employed either NADH–haem distances, or double phe‐gate conformations. NADH–haem distances were determined from the Cartesian distance between the *β*‐*meso*‐carbon in haem and the hydride‐bearing carbon in NADH within each minimum. For phe‐gate conformations, the phenylalanine (whether F104 or F199) was always defined with reference to the haem molecule. Specifically, a dihedral angle, θ, between phenylalanine and haem was defined according to the α, γ, and ζ carbon atoms of the residue and the *β*‐*meso*‐carbon of haem, in the order C*α*‐C*γ*‐Cζ‐C*β*‐*meso*. The four possible conformations for the double phe‐gate were then assigned according to the following principles, where CC is closed‐closed, CO is closed‐opened, and so on:

- CC: –90° ≤ *θ*_F104_ < 80° and 0° ≤ *θ*_F199_ < 175°.
- CO: –90° ≤ *θ*_F104_ < 80° and (*θ*_F199_ < 0° or *θ*_F199_ ≥ 175°).
- OC: (*θ*_F104_ < −90° or *θ*_F104_ ≥ 80°) and 0° ≤ *θ*_F199_ < 175°.
- OO: (*θ*_F104_ < −90° or *θ*_F104_ ≥ 80°) and (*θ*_F199_ < 0° or *θ*_F199_ ≥ 175°).

## AUTHOR CONTRIBUTIONS

**Alasdair D. Keith:** Conceptualization; methodology; software; validation; formal analysis; investigation; data curation; writing – original draft; writing – review and editing; visualization. **Elizabeth B. Sawyer:** Conceptualization; methodology; validation; formal analysis; investigation; writing – review and editing. **Desmond C. Y. Choy:** Conceptualization; methodology; software; formal analysis; investigation; data curation; writing – review and editing. **James L. Cole:** Investigation. **Cheng Shang:** Methodology; investigation. **George S. Biggs:** Methodology; investigation. **Oskar James Klein:** Methodology; investigation. **Paul D. Brear:** Investigation; data curation; visualization. **David J. Wales:** Conceptualization; methodology; software; formal analysis; resources; writing – review and editing; supervision; project administration; funding acquisition. **Paul D. Barker:** Conceptualization; methodology; software; formal analysis; resources; writing – review and editing; supervision; project administration; funding acquisition.

## CONFLICT OF INTEREST STATEMENT

The authors declare no conflicts of interest.

## Supporting information


**Data S1.** Supporting Information.
