# Supplementary material for: Investigation into the effect of phenylalanine gating on anaerobic haem breakdown using the energy landscape approach
Source: Protein Sci. 2025 Jan 28;34(2):e5243. doi: 10.1002/pro.5243 (PMC11773379; doi:10.1002/pro.5243)
Supplement: Supplementary file 1 — Data S1. Supporting Information. [file PRO-34-e5243-s001.pdf]

Investigation into the Effect of Phenylalanine Gating on Anaerobic Haem Breakdown using the Energy Landscape Approach

Alasdair D. Keith,[*,†] Elizabeth B. Sawyer,[†] Desmond C.Y. Choy,[†] James L. Cole,[†] Cheng Shang,[†] George S. Biggs,[†] Oskar James Klein,[†] Paul D. Brear,[‡] David J. Wales,[*,†] and Paul D. Barker[*,†]

†*Yusuf Hamied Department of Chemistry, University of Cambridge, Cambridge CB2 1EW, United Kingdom*

‡*Department of Biochemistry, Sanger Building, University of Cambridge, Cambridge CB2 1GA, United Kingdom*

E-mail: alasdairkeithrfc@gmail.com; dw34@cam.ac.uk; pdb30@cam.ac.uk

A Ligand Structural Formulae

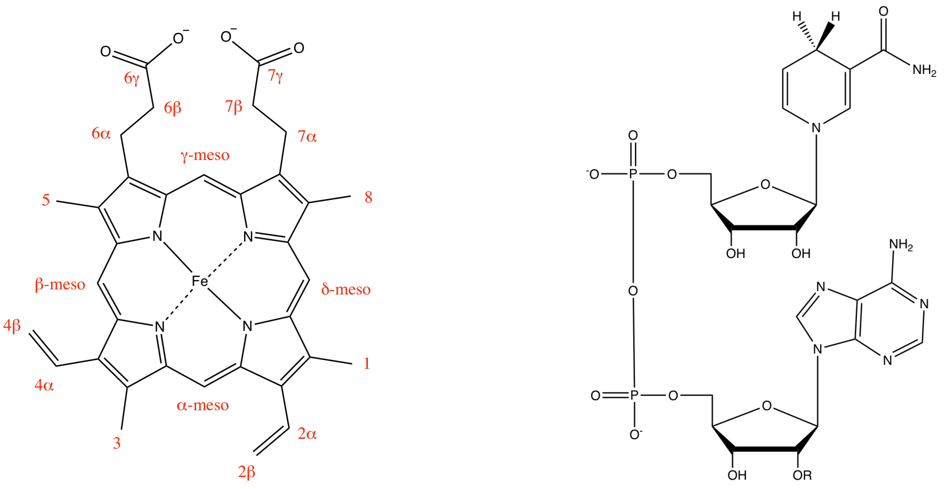

Figure S1: Left: Labelled haem b structural formula. Right: NAD(P)H structural formula. The representation is in a folded conformation, which is its natural state in aqueous solution. Those hydrides that can be transferred over to haem are explicitly highlighted, and shown as pointing in/out of the plane. R = H gives NADH; R = PO_3 gives NADPH. NADPH can also effect haem breakdown to give the haem breakdown product (HBP), but NADH is the ligand studied in detail in the present work.

B HemS Genetic Sequence

The dnaseq package[1] was used to represent the genetic sequence of HemS.

```
   1 atgagcaaat caatatacga gcagtatcta caagctaaag cagataatcc
  51 gggcaaatat gcgcgcgatt tggccacgct gatggggatt tcagaagcgg
 101 aactgaccca tagccgcgtt agtcatgatg ccaaacgtct gaaaggtgat
 151 gcccgcgcac tactggccgc attggaagct gtcggtgagg tcaaagctat
 201 cacccgcaac acctatgccg tacatgagca aatgggccgt tatgaaaatc
 251 aacatctgaa tggccatgct ggtttgatcc tcaatccacg caatttagat
 301 ttacgcctgt tcctcaacca gtgggccagc gcattcacgc tgacagaaga
 351 aactcgccac ggtgtacgcc atagcatcca gtttttcgac catcaaggcg
 401 atgctctgca taaagtgtat gtcactgaac aaactgacat gccagcctgg
 451 gaagcgctac tggcgcagtt tatcaccaca gaaaatccag agttacagct
 501 agagccactg agcgcacctg aagtcactga accgacagcc accgatgaag
 551 ctgtcgatgc tgaatggcgt gctatgactg acgtgcatca gttcttccag
 601 ttgctcaaac gcaataattt gacccgtcag caagccttcc gtgccgtggg
 651 taatgatctg gcttatcagg ttgataacag ttctctgacc cagttactga
 701 acattgctca gcaagaacag aatgaaatca tgatttttgt gggtaaccgt
 751 ggctgtgtac aaatattcac cggcatgatt gaaaaggtta caccacatca
 801 agattggatt aatgttttca accagcgctt cacgctgcat ctgattgaaa
 851 caacgattgc tgaaagctgg attacccgca agccaacaaa agacggtttc
 901 gtgaccagtt tggaactgtt tgctgctgat ggcacccaaa ttgcacaact
 951 ttacggtcag cgcaccgaag gccagccaga acaaacgcaa tggcgtgagc
1001 aaattgctcg cctcaataat aaggatatcg ccgcatga
```

C HemS Primers

The primers used for point-mutations are displayed below. Sequences are all given from 5′ to 3′, with mutagenic bases capitalised.

F104A

Forward Sequence: agatttacgcctgGCcctcaaccagtggg

Reverse Complement: cccactggttgaggGCcaggcgtaaatct

F104I

Forward Sequence: agatttacgcctgAtcctcaaccagtgggc

Reverse Complement: gcccactggttgaggaTcaggcgtaaatct

F199A

Forward Sequence: gactgacgtgcatcagttcGCccagttgctcaaacgc

Reverse Complement: gcgtttgagcaactggGCgaactgatgcacgtcagtc

D HemS Protein Sequence

There were small points of differentiation between the computational and experimental sequences used. The N-terminal (MSK) and C-terminal (DIAA) residues were removed in the computational sequence to simplify computations. These residues, being far from the protein cavity, and not appearing in any experimental crystal structures, were not considered important for protein structure or function.

Residue 333 was glutamic acid in the computational sequence rather than aspartic acid; this mutation actually causes the computational sequence to be closer to the published structure for HemS (PDB: 2J0P).[2] We believe that the gene provided by Schneider and Paoli[2,3] had mutated at this position, causing all expressed HemS in this work to contain the incorrect glutamic acid. As this residue is located in a loop at the surface, we believe that it should have little influence on function.

The last point of differentiation is at residue 197, where the glutamine found in the experimental sequence has been converted to a glutamic acid. This mutation is due to a mistaken assignment in PDB structure 2J0P,[2] which was used as the starting structure for our computational calculations. The discrepancy was only revealed after our research program concluded. Test calculations, detailed in Keith *et al.*,[4] have shown that, although this residue is situated close to the main cavity, it does not have any significant effect on the protein structure or on the thermodynamics of the interplay between HemS, haem and NADH. Those residues that differ between the experimental and computational sequences are highlighted in red, and those selected for mutation are highlighted in green.

HemS – Protein Sequence (Experimental)

```
  1 MSKSIYEQYL QAKADNPGKY ARDLATLMGI SEAELTHSRV SHDAKRLKGD
 51 ARALLAALEA VGEVKAITRN TYAVHEQMGR YENQHLNGHA GLILNPRNLD
101 LRLFLNQWAS AFTLTEETRH GVRHSIQFFD HQGDALHKVY VTEQTDMPAW
151 EALLAQFITT ENPELQLEPL SAPEVTEPTA TDEAVDAEWR AMTDVHQFFQ
201 LLKRNNLTRQ QAFRAVGNDL AYQVDNSSLT QLLNIAQQEQ NEIMIFVGNR
251 GCVQIFTGMI EKVTPHQDWI NVFNQRFTLH LIETTIAESW ITRKPTKDGF
301 VTSLELFAAD GTQIAQLYGQ RTEGQPEQTQ WREQIARLNN KDIAA
```

E Protein Masses and Purities

Following expression, protein masses were checked by mass spectrometry, and their purities by SDS-PAGE.

Table S1: WT and mutant accurate masses. Actual masses were determined by mass spectrometry.

Protein	Expected Mass / Da	Actual Mass / Da	Difference / Da
**Wild Type**	39,360	39,360	0
**F104A**	39,284	39,284	0
**F104AF199A**	39,208	39,208	0
**F104I**	39,326	39,326	0
**F199A**	39,284	39,284	0

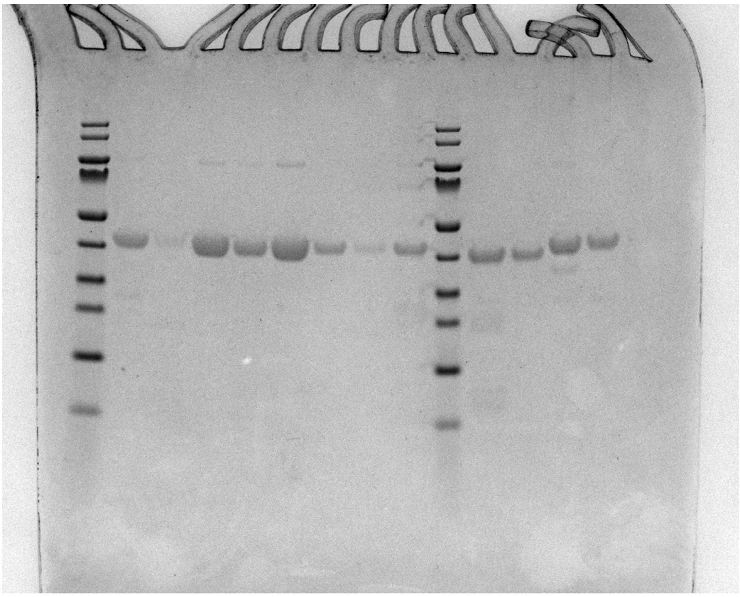

Figure S2: SDS-PAGE gel of the WT and mutant HemS proteins studied in this work, alongside further mutants and selected homologues. The His-tags have been removed in all instances. Lane numbering is from left-to-right. Lanes 1 and 10 are occupied by PageRuler Protein Ladder markers, with bands ranging from 10 kDa to 180 kDa. The samples are all shown to have high levels of purity. Lane 2: WT HemS; 3: F104A HemS; 4: F104AF199A HemS; 5: F104I HemS; 6: F199A HemS; 7: R209A HemS; 8: R209K HemS; 9: Q210A HemS; 11: WT ChuS; 12: WT ShuS; 13: WT HmuS; 14: WT HemS (repeated).

F Conservations of Key Residues

Table S2: Sequence conservation of selected residues. 218 homologues from 218 different genera were compared to provide the data. Residue indexing is according to their respective positions within HemS. Residue 318 is a tyrosine in HemS, but is a phenylalanine across 69.7% of the homologues. The double phenylalanine gate showed remarkable levels of conservation given F104 and F199 are not obviously involved in ligand-binding. The % conservation of F199 rises to 90.8% when the other aromatic residues (histidine and tyrosine) are considered. Table reproduced from Keith *et al.*[4]

**Function**	**Residue**	**% Conservation**
Haem-binding	R102	100%
Haem-binding	H196	100%
Haem-binding	R209	100%
Haem-binding	K294	98.1%
Haem-binding	F318	69.7%
Haem-binding	R321	99.1%
NADH-binding	Q132	29.8%
NADH-binding	K203	43.1%
NADH-binding	R250	61.5%
NADH-binding	T312	30.7%
Phe-gate	F104	98.6%
Phe-gate	F199	82.6%

G Phylogenetic Tree

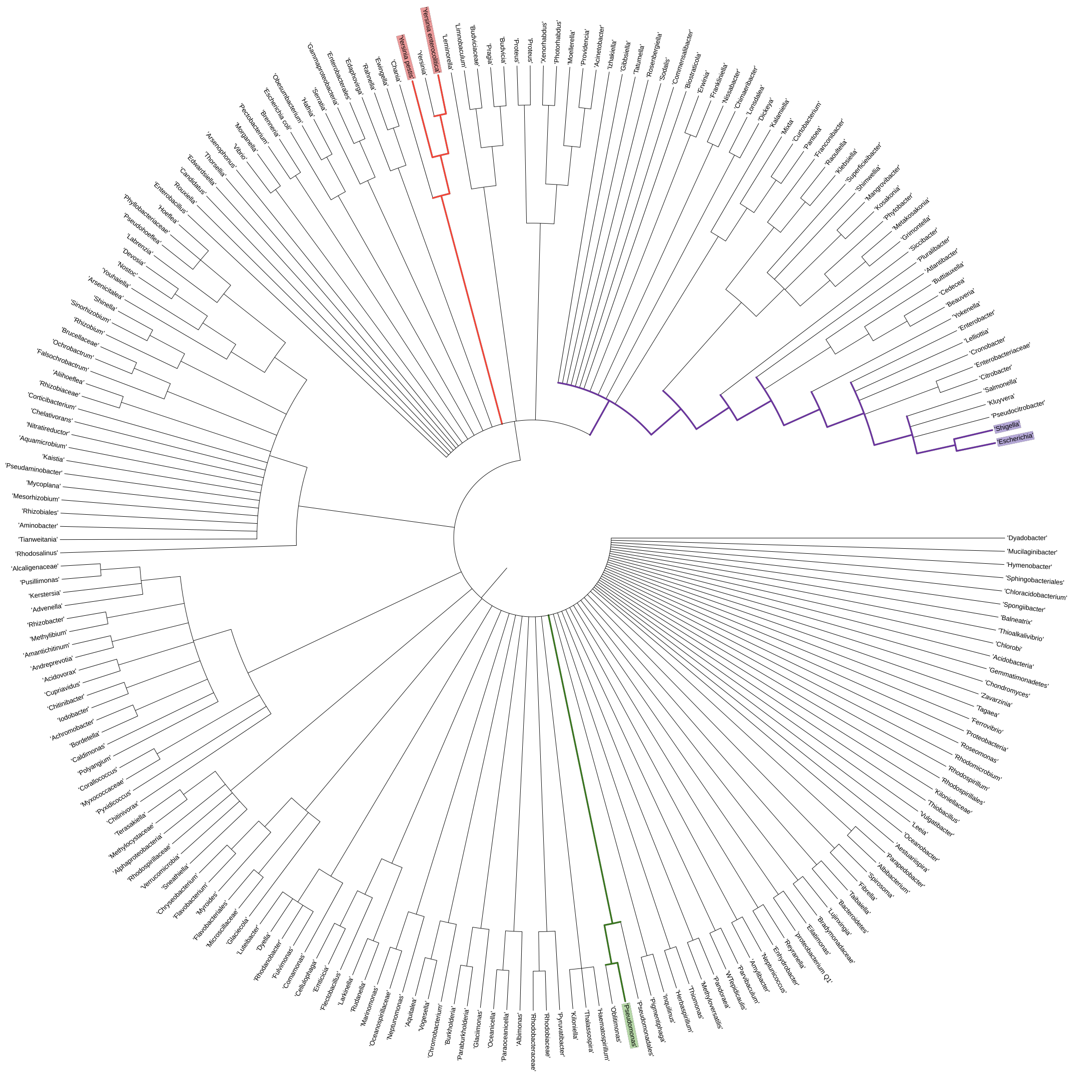

Figure S3: Maximum likelihood phylogenetic tree for HemS and its homologues. 218 different genera are represented, including both pathogenic and non-pathogenic bacteria. Selected species are highlighted. Red: *Yersinia enterocolitica* (HemS), *Yersinia pestis* (HmuS). Purple: *Escherichia coli* (ChuS), *Shigella dysenteriae* (ShuS). Green: *Pseudomonas aeruginosa* (PhuS). Figure and caption reproduced from Keith *et al.*[4]

H WT and Mutant UV-Visible Haem-Binding Spectra

Haem-binding was tested at pH values 5.0, 6.5 and 8.0. The protein samples typically precipitated out of solution at pH 5.0. The F104A sample degraded before running these experiments, and so was excluded.

Fig. S4 shows the spectra for experiments run at pH 8.0. Mutations do not significantly disrupt any of the Soret peaks, implying that the nature of haem-binding does not significantly change upon changes to the phe-gate. Molar absorbance is slightly reduced upon mutation, suggesting the strength of haem-binding is marginally reduced.

Our previous research[4] on WT HemS and its homologues (HmuS, ChuS and ShuS) showed that the wavelength and intensity of the Soret band typically increase as the pH increases. F104AF199A and F199A proved to be exceptions to this trend, with ϵ_{SM} decreasing or effectively staying the same upon an increase in pH.

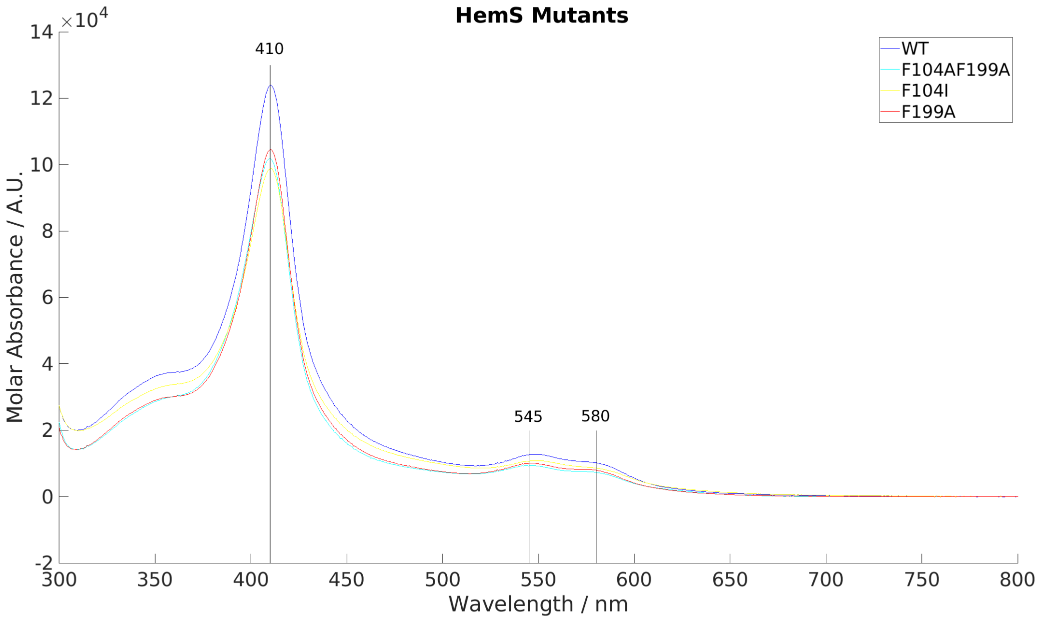

Figure S4: WT and mutant UV-Visible haem-binding spectra at pH 8.0. The α-, β- and Soret bands are highlighted.

Table S3: WT and mutant haem-binding properties. The Soret maximum is given in nm, and ϵ_{SM} is the extinction coefficient in $M^{-1}\,cm^{-1}$ at these quoted Soret maxima.

	**pH 6.5**		**pH 8.0**	
	**Soret Max.**	ϵ_{SM}	**Soret Max.**	ϵ_{SM}
**Wild Type**	409.00	103,000	410.25	123,900
**F104AF199A**	406.50	104,300	409.75	101,800
**F104I**	408.50	93,900	410.50	98,800
**F199A**	408.00	104,400	410.50	104,500

I WT and Mutant UV-Visible Reaction Spectra

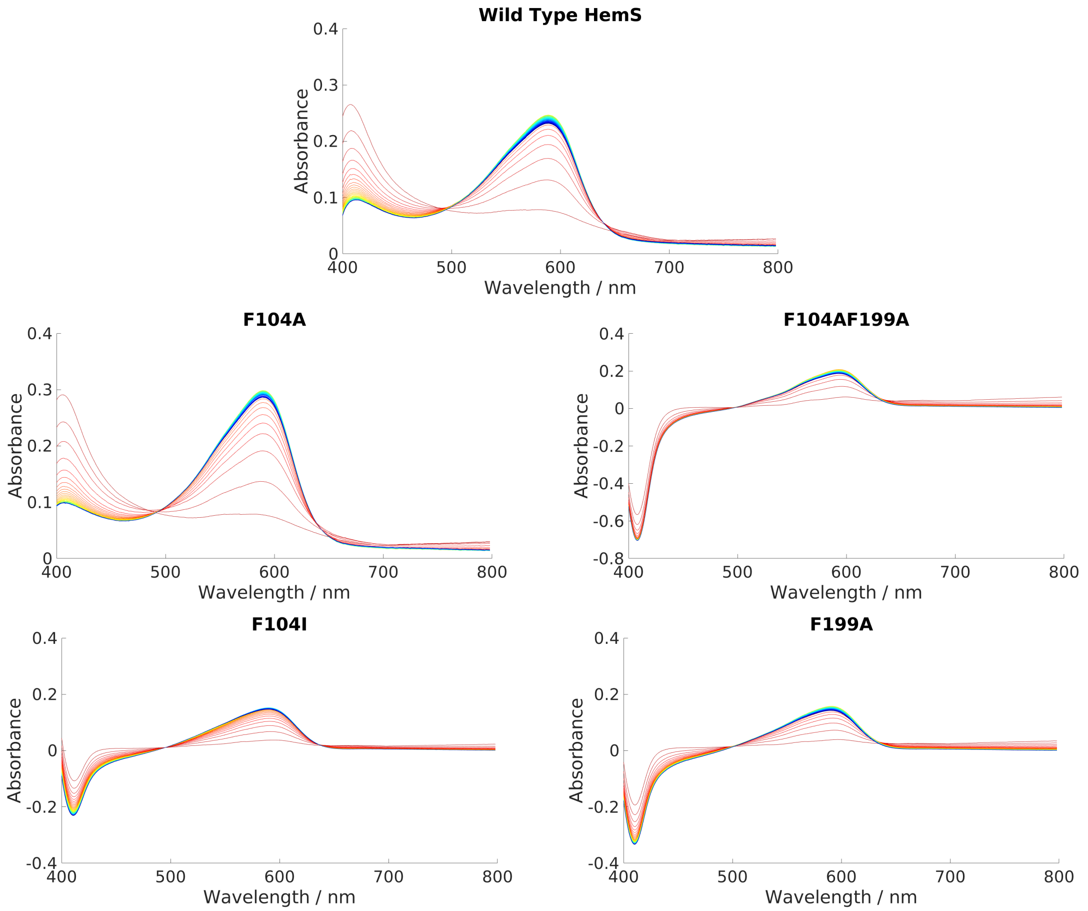

Figure S5: WT and mutant UV-Visible spectra. In each case, reaction stoichiometry was 8 μM protein : 8 μM haem : 2000 μM NADH. Scans were run for 50 minutes in one minute increments, starting at red (1 minute) and moving through to blue/purple (50 minutes). These experiments were run before the importance of HemS : haem pre-equilibration was appreciated. Therefore, positive absorbance values between 400 and 450 nm for the WT and F104A HemS samples were detected, which indicates that haem had not properly equilibrated before the baseline was taken. These results, whilst not quantitatively accurate, show *via* growth in absorbance at 591 nm that all of the mutants are capable of engaging in the haem breakdown reaction.

J Crystallographic Data

Table S4: F104AF199A Crystallography Statistics

**PDB code**	**7QXV**
**Data Collection**	
Date of collection	01/06/2021
Beamline	Diamond beamline I04
Wavelength	0.9793
Resolution range	50.57 - 1.67 (1.70 - 1.67)
Space group	P 21 21 21
Unit cell	62.03 69.613 73.77 90 90 90
Total reflections	701834 (32887)
Unique reflections	37692 (1840)
Multiplicity	18.6 (17.9)
Completeness (%)	100.0 (100.0)
Mean I/sigma(I)	5.6 (0.2)
Wilson B-factor	37.22
R-merge	0.40 (91.25)
R-meas	0.42 (93.93)
R-pim	0.10 (22.14)
CC1/2	0.99 (0.34)
**Refinement**	
Resolution range	50.57 - 1.67 (1.71 - 1.67)
Reflections used in refinement	32065 (484)
Reflections used for R-free	1668 (35)
R-work	0.23 (0.70)
R-free	0.28 (0.87)
Completeness (%)	89.5 (18.36)
Number of non-hydrogen atoms	2843
-macromolecules	2737
-ligands	7
-solvent	99
Protein residues	339
RMS(bonds)	0.013
RMS(angles)	1.85
Ramachandran favored (%)	94.66
Ramachandran allowed (%)	5.34
Ramachandran outliers (%)	0
Rotamer outliers (%)	2.41
Clashscore	4.6
Average B-factor	54.01
-macromolecules	54.25
-ligands	54.53
-solvent	47.18

K Small Molecule Electron Density from Crystallography

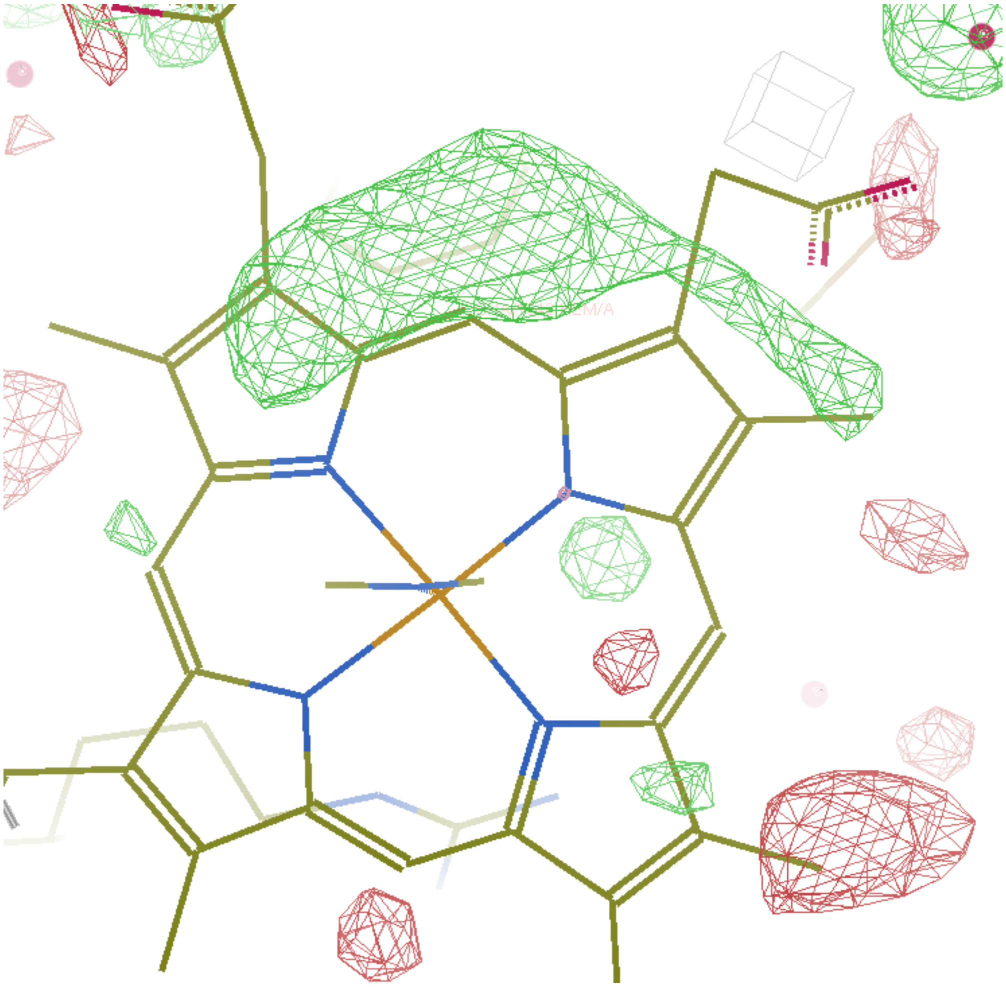

Figure S6: The difference electron density map contoured at 3 sigma for the structure of F104AF199A, with a representative heme molecule overlaid. Attempts had been made to crystallise the protein structure with the HBP intact and inside the pocket. However, addition of cryoprotectant resulted in the characteristic purple colour (which is caused by the electronic properties of the HBP itself) of the crystals seeping into the surrounding medium, thus suggesting fragmentation and expulsion of the HBP. The density clearly does not correspond to a break down product of haem, thus confirming that the ligand was expelled from the pocket.

References

(1) Pedersen, B. The dnaseq package. 2002.

(2) Schneider, S.; Sharp, K. H.; Barker, P. D.; Paoli, M. An induced fit conformational change underlies the binding mechanism of the heme transport proteobacteria-protein HemS. *J. Biol. Chem.* **2006**, *281*, 32606–32610.

(3) Schneider, S.; Paoli, M. Haem-binding properties and crystallisation of the bacterial protein HemS. *Acta Cryst. A* **2005**, *61*, 343.

(4) Keith, A. D.; Sawyer, E. B.; Choy, D. C. Y.; Xie, Y.; Biggs, G. S.; Klein, J.; Brear, P. D.; Wales, D. J.; Barker, P. D. Combining experiment and energy landscapes to explore anaerobic heme breakdown in multifunctional hemoproteins. *Phys. Chem. Chem. Phys.* **2023**, *26*, 695–712.
